# Supplementary material for: A randomized controlled trial of heterologous ChAdOx1 nCoV-19 and recombinant subunit vaccine MVC-COV1901 against COVID-19
Source: Nat Commun. 2022 Sep 17;13:5466. doi: 10.1038/s41467-022-33146-7 (PMC9482645; doi:10.1038/s41467-022-33146-7)
Supplement: Supplementary file 1 — Supplementary information [file 41467_2022_33146_MOESM1_ESM.pdf]

Supplementary Information File

Supplementary Table 1. Summary of demographics and clinical characteristics.

Characteristic	Total		ChAdOx1/ MVC-COV1901		ChAdOx1/ ChAdOx1		P#
	n	(%)	n	(%)	n	(%)	
**Demographics**							
Age, years							0.991
Mean ± SD	100	40.9 ± 8.8	50	40.9 ± 9.0	50	40.9 ± 8.7	
Median, range		40, 22-62		40, 22-59		40, 24-62	
Sex							0.689
Male	50	(50.0)	24	(48.0)	26	(52.0)	
Female	50	(50.0)	26	(52.0)	24	(48.0)	
Body height, cm, Mean ± SD	100	165.7 ± 8.2	50	165.2 ± 8.1	50	166.2 ± 8.4	0.559
Body weight, kg, Mean ± SD	100	67.5 ± 14.8	50	66.2 ± 15.6	50	68.9 ± 14.1	0.367
Heart Rate, beat/min, Mean ± SD	100	87.1 ± 14.5	50	87.9 ± 14.6	50	86.4 ± 14.6	0.609
SBP, mmHg, Mean ± SD	100	124.9 ± 17.9	50	122.8 ± 16	50	126.9 ± 19.5	0.253
DBP, mmHg, Mean ± SD	100	81.3 ± 11.2	50	79.6 ± 9.1	50	83 ± 12.9	0.129
BT, ˚C, Mean ± SD	100	36.5 ± 0.4	50	36.5 ± 0.4	50	36.6 ± 0.3	0.148
**Baseline hemogram values**							
Hemoglobin, g/dL, Mean ± SD	100	13.8 ± 1.7	50	13.8 ± 1.6	50	13.8 ± 1.9	0.923
WBC count, per µL, Mean ± SD	100	6.3 ± 1.8	50	6.2 ± 1.9	50	6.5 ± 1.7	0.342
Platelet count, per µL, Mean ± SD	100	261.3 ± 63.3	50	262.9 ± 49.7	50	259.8 ± 74.9	0.807
**Baseline biochemistry values**							
BUN, mEq/L, Mean ± SD	100	12.8 ± 3.1	50	13.2 ± 3.6	50	12.4 ± 2.5	0.201

Creatinine, mEq/L, Mean ± SD	100	0.8 ± 0.2	50	0.8 ± 0.2	50	0.8 ± 0.2	0.501
ALT, U/L, Mean ± SD	100	27.5 ± 24.3	50	26.1 ± 24	50	28.9 ± 24.7	0.567
AST, U/L, Mean ± SD	100	20.9 ± 22.2	50	18.5 ± 7.8	50	23.3 ± 30.3	0.286
CRP, mg/L, Mean ± SD	100	1.7 ± 2.2	50	1.6 ± 2.3	50	1.8 ± 2.2	0.658
**Major medical history**							
Type 2 diabetes mellitus	3	(3.0)	2	(4.0)	1	(2.0)	>0.999
Goiter	1	(1.0)	0	(0.0)	1	(2.0)	>0.999
Hypothyroidism	2	(2.0)	1	(2.0)	1	(2.0)	>0.999
**Dose interval**							
Days, Mean ± SD	100	50.6 ± 13.7	50	50.7 ± 13.3	50	50.6 ± 14.3	0.965
No. for 4-6 weeks interval (%)	50	(50.0)	25	(50.0)	25	(50.0)	
No. for 8-10 weeks interval (%)	50	(50.0)	25	(50.0)	25	(50.0)	

Abbreviations. SD, standard deviation; SBP, systolic blood pressure; Diastolic blood pressure, DBP; body temperature, BT; white blood cells count, WBC; C-reactive protein, CRP.

[#] P values were determined by t test for the continuous variables and chi-square test for the categorical variables (two-tailed).

Supplementary Table 2. Medical history of study participants.

Disease	ChAdOx1/ MVC-COV1901	ChAdOx1/ ChAdOx1	Total
ACUTE ATOPIC CONJUNCTIVITIS, BILATERAL	1	0	1
ACUTE GINGIVITIS, PLAQUE INDUCED	1	0	1
ACUTE NASOPHARYNGITIS (COMMON COLD)	0	1	1
ACUTE SINUSITIS	0	1	1
ACUTE SINUSITIS, UNSPECIFIED	0	1	1
ACUTE UPPER RESPIRATORY INFECTION	1	0	1
ACUTE VAGINITIS	1	0	1
ADVERSE EFFECT OF OTHER VIRAL VACCINES, INITIAL ENCOUNTER.	1	0	1
AGRESSIVE PERIODONTOSIS, LOCALIZED	0	1	1
ALLERGIC RHINITIS, UNSPECIFIED	0	2	2
ALT INCRTEASRD GR.2	0	1	1
ANEMIA, UNSPECIFIED	0	1	1
ANGINA PECTORIS, UNSPECIFIED	0	1	1
AST INCREASED GR.3	0	1	1
ATTENTION DEFICIT HYPERACTIVITY	0	1	1
BEMIGN PAROXYSMAL VERTIGO	0	1	1
BENIGN PROSTATIC HYPERPLASIA WITH LOWER URINARY TRACT SYMPTOMS	1	0	1
CELLULITIS OF LEFT ORBIT	0	1	1
CEREBRAL INFARCTION DUE TO THROMBOSIS OF UNSPECIFIED CEREBRAL ARTERY	0	1	1
CHEST PAIN	0	1	1
CHONDROMALACIA PATELLAE, UNSPECIFIED KNEE	1	0	1
CHRONIC APICAL PERIODONTITIS	1	0	1
CHRONIC FATIGUE, UNSPECIFIED	1	0	1
CHRONIC GIANT PAPILLARY CONJUNCTIVITIS, RIGHT EYE	0	1	1
CHRONIC GINGIVITIS, PLAQUE INDUCED	0	1	1
CHRONIC PEPTIC ULCER, SITE UNSPECIFIED, WITHOUT HEMORRHAGE OR PERFORATION	0	1	1
CHRONIC PERIDONTITIS, GENERALIZED	1	0	1
CONTUSION OF RIGHT LOWER LEG, INITIAL ENCOUNTER	1	0	1
CONTUSION OF UNSPECIFIED FOREARM, INITIAL ENCOUNTER	1	0	1
CONTUSION OF UNSPECIFIEDWRIST, INITIAL ENCOUNTER	0	1	1
DENTAL CARIES, UNSPECIFIED	1	0	1
DENTIAL CARIES, UNSPECIFIED	1	0	1

DENTIAL ROOT CARIES	1	0	1
DISEASES OF THE SKIN AND SUBCUTANEOUS TISSUE	1	0	1
DISORDER OF THYROID, UNSPECIFIED.	1	0	1
DIZZINESS AND GIDDINESS	0	1	1
DYSMENORRHEA	0	1	1
DYSTHYMIC DISORDER	0	2	2
ESSENTIAL(PRIMARY)HYPERTENSION	2	1	3
FECAL IMPACTION	0	1	1
FURUNCLE, UNSPECIFIED	1	1	2
GASTRIC ULCER, UNSPECIFIED AS ACUTE OR CHRONIC, WITHOUT HEMORRHAGE OR PERFORATION	1	0	1
GENERALIZED ANXIETY DISORDER.	1	0	1
GLAUCOMA SECONDARY TO OTHER EYE DISORDERS, RIGHT EYE, STAGE UNSPECIFIED	0	1	1
HORDEOLUM EXTERNUM LEFT UPPER EYELID	0	1	1
HORDEOLUM EXTERNUM UNSPECIFIED EYE, UNSPECIFIED EYELID	0	1	1
HYPERTENSION	2	4	6
HYPERTENSIVE HEART DISEASE WITHOUT HEART FAILURE	2	0	2
HYPOTHYROIDISM, UNSPECIFIED.	1	0	1
HYPOTHYROIDISM, UNSPECIFIED	0	1	1
IDIOPATHIC URTICARIA	0	1	1
INSOMINA	1	0	1
INTERAL CAROTID ARTERIES, BILATERAL	1	0	1
IRON DEFICIENCY ANEMIA, UNSPECIFIED	0	1	1
IRRITABLE BOWEL SYNDROME WITH DIARRHEA	0	1	1
LEIOMYOMA OF UTERUS	0	1	1
LEIOMYOMA OF UTERUS, UNSPECIFIED.	1	0	1
LOCAL INFECTION, SUBCUTANEOUS TISSUE, UNSPECIFIED	0	1	1
LOWER ABDOMINAL PAIN, UNSPECIFIED	1	0	1
MAJOR DEPRESSIVE DISORDER, RECURRENT, IN REMISSION, UNSPECIFIED	0	1	1
MYALGIA	1	0	1
NONINFECTIVE GASTROENTERITIS AND COLITIS, UNSPECIFIED	1	0	1
OTHER CHRONIC CONJUNCTIVITIS	0	1	1
OTHER DISEASES OF STOMACH AND DUODENUM	0	1	1
OTHER SPECIFIED DERMATITIS	2	0	2
OTHER SPECIFIED NONINFECTIVE GASTROENTERITIS AND COLITIS	1	0	1

OTHER SPECIFIED NONINFECTIVE GASTROENTERITIS AND COLITIS.	1	0	1
OTHER SPECIFIED NONINFECTIVE GASTROENTERITIS COLITIS	0	1	1
OTHER SPONDYLOSIS WITH MYELOPATHY, CERVIAL REGION	0	1	1
PERIODONTITIS	1	0	1
PERIODONTOSIS	1	1	2
POLYCYSTIC OVARIAN SYNDROME	0	1	1
POSTMENOPAUISAL ATROPHIC VAGINITIS	1	0	1
PRIMARY INSOMNIA	0	1	1
PRURITUS, UNSPECIFIED	1	0	1
PURE HYPERCHOLESTEROLEMIA	1	0	1
RETAINED DENTAL ROOT	0	1	1
SCAR CONDITIONS AND FIBROSIS OF SKIN	0	1	1
THYROTOXICOSIS EITH DIFFUSE GOITER WITHOUT THYROTOXIC CRISIS OR STORM	0	1	1
TOXIC EFFECT OF VENOM OF OTHER ARTHROPOD, ACCIDENTAL (UNINTENTIONAL), SUBSEQUENT ENCOUNTER	1	0	1
TRULICITY	0	1	1
TYPE 2 DIABETES MELLITUS WITHOUT COMPLICATIONS	2	1	3
UNSPECIFIED ASTHMA, UNCOMPLICATED	1	0	1
UNSPECIFIED CONJUNCTIVITIS	1	0	1
UNSPECIFIED CONTACT DERMATITIS	2	0	2
UNSPECIFIED EUSTACHIAN TUBE DISORDER	0	1	1
UNSPECIFIED INTERNALDERANGEMENT OF UNSPECIFIED KNEE	1	0	1
UNSPECIFIED OBSTRUCTION OF EUSTACHIAN TUBE, UNSPECIFIED EAR	0	1	1
UNSPECIIFIED OTITIS EXTERNA, UNSPECIFIED EAR	1	0	1
UPPER ABDOMINAL PAIN, UNSPECIFIED	0	1	1
URTICARIA, UNSPECIFIED	1	0	1

Supplementary Table 3. Summary of solicited local and systemic adverse events within 7 days of booster vaccination.

Adverse events		Total (N=100)		ChAdOx1/ MVC-COV1901 (N=50)		ChAdOx1/ ChAdOx1 (N=50)		P#
Term	Grade	n	(%)	n	(%)	n	(%)	
**Local**								
Erythema	Any	10	(10.0)	6	(12.0)	4	(8.0)	
	1	9	(9.0)	5	(10.0)	4	(8.0)	
	2	1	(1.0)	1	(2.0)	0	(0.0)	>0.999
Pain	Any	68	(68.0)	35	(70.0)	33	(66.0)	
	1	62	(62.0)	33	(60.0)	29	(54)	
	2	3	(3.0)	2	(4.0)	1	(2.0)	
	3	3	(3.0)	0	(0.0)	3	(6.0)	0.421
Swelling	Any	14	(14.0)	7	(14.0)	7	(14.0)	
	1	13	(13.0)	6	(12.0)	7	(14.0)	
	2	1	(1.0)	1	(2.0)	0	(0.0)	0.273
**Systemic**								
Diarrhea	Any	11	(10.0)	3	(6.0)	8	(16.0)	
	1	10	(10.0)	2	(4.0)	8	(16.0)	
	2	1	(1.0)	1	(2.0)	0	(0.0)	0.273
Fatigue	Any	46	(46.0)	24	(48.0)	21	(42.0)	
	1	34	(34.0)	21	(42.0)	13	(26.0)	
	2	10	(10.0)	2	(4.0)	8	(16.0)	

	3	2	(2.0)	1	(2.0)	1	(2.0)	0.028
Fever	1	2	(2.0)	2	(4.0)	0	(0.0)	NA
Headache	Any	30	(30.0)	14	(28.0)	16	(32.0)	
	1	25	(25.0)	13	(26.0)	12	(24.0)	
	2	4	(4.0)	1	(2.0)	3	(6.0)	
	3	1	(1.0)	0	(0.0)	1	(2.0)	0.336
Myalgia	Any	27	(27.0)	14	(28.0)	16	(32.0)	
	1	25	(25.0)	12	(24.0)	13	(26.0)	
	2	4	(4.0)	1	(2.0)	3	(6.0)	
	3	1	(1.0)	1	(2.0)	0	(0.0)	>0.999
Vomiting	Any	6	(6.0)	3	(6.0)	3	(6.0)	
	1	5	(5.0)	3	(6.0)	2	(4.0)	
	2	1	(1.0)	0	(0.0)	1	(2.0)	>0.999

# P values were determined by Chi-square test or Fisher's exact test as appropriate (two-tailed).

Supplementary Table 4. Antibody geometric mean titers against SARS-CoV-2 prior to and after booster vaccination.

	ChAdOx1/ MVC-COV1901		ChAdOx1/ ChAdOx1			
	n	GMT (95% CI)	n	GMT (95% CI)	GMT ratio (95% CI)	P[#]
nAb by ELISA method (IU/mL)						
Day 0	50	32.2 (22.8, 45.6)	50	30.2 (21.6, 42.3)	1.1 (0.7, 1.7)	0.790
Day 10±3	50	202.1 (162.1, 252.1)	50	77.9 (53.9, 112.5)	2.6 (1.7, 4.0)	<0.001
Day 28±3	50	235.5 (186.7, 297.1)	50	114.8 (86.7, 152.1)	2.1 (1.4, 2.9)	<0.001
nAb against variants at day 28±3 (NT_{50})						
Wuhan variant	50	42.9 (35.1, 52.6)	50	17.1 (13.0, 22.5)	2.5 (1.8, 3.5)	<0.001
Delta variant	50	51.3 (39.8, 65.9)	50	19.8 (14.7, 26.8)	2.6 (1.8, 3.8)	<0.001
bAb against S1 protein (ELISA Unit)						
Day 0	50	4.9 (4.1, 5.9)	50	4.5 (3.9, 5.3)	1.1 (0.8, 1.4)	0.526
Day 10±3	50	14.2 (11.4, 17.8)	50	7.3 (5.8, 9.2)	2.0 (1.4, 2.7)	<0.001
Day 28±3	50	15.9 (13.1, 19.3)	50	8.2 (6.8, 9.9)	1.9 (1.5, 2.5)	<0.001
bAb against RBD (ELISA Unit)						
Day 0	50	4.0 (3.5, 4.6)	50	3.8 (3.4, 4.2)	1.1 (0.9, 1.3)	0.485
Day 10±3	50	7.8 (6.5, 9.5)	50	5.2 (4.4, 6.2)	1.5 (1.2, 1.9)	0.002
Day 28±3	50	9.4 (7.7, 11.4)	50	5.3 (4.6, 6.2)	1.8 (1.4, 2.2)	<0.001

Abbreviations: GMT, geometric mean titer; CI, confidence interval; nAb, neutralizing antibody; bAb, binding antibody; RBD, receptor-binding domain

[#] P values were determined by t test (two-tailed).

Supplementary Table 5. Correlation matrix (Pearson correlation coefficients[#]) for antibody immune response.

	ELISA-based nAb	nAb against Wuhan	nAb against Delta variant	bAb to S1	bAb to RBD
ELISA-based nAb	1	0.718 (0.608, 0.802) P < 0.001	0.663 (0.536, 0.760) P < 0.001	0.862 (0.801, 0.905) P < 0.001	0.786 (0.698, 0.851) P < 0.001
nAb against Wuhan		1	0.840 (0.771, 0.890) P < 0.001	0.767 (0.672, 0.837) P < 0.001	0.696 (0.579, 0.785) P < 0.001
nAb against Delta variant			1	0.697 (0.580, 0.786) P < 0.001	0.631 (0.497, 0.736) P < 0.001
bAb to S1				1	0.944 (0.918, 0.962) P < 0.001
bAb to RBD					1

Abbreviations. nAb, neutralizing antibody; bAb, binding antibody; RBD, receptor-binding domain.

[#] The Pearson method was used to calculate the coefficients of correlations and the significance of the correlations.

Supplementary Table 6. Geometric mean concentrations (IU/mL) of neutralizing antibody to SARS-CoV-2 by dose interval.

Days post boosting	4-6 weeks interval*		8-10 weeks interval*			
	n	GMT (95% CI)	n	GMT (95% CI)	GMT ratio (95% CI)	P[#]
MVC-COV1901						
Day 0	25	39.0 (23.5, 64.8)	25	26.6 (16.2, 43.7)	1.5 (0.7, 2.9)	0.272
Day 10±3	25	258.4 (208.4, 320.3)	25	158.2 (108.5, 230.6)	1.6 (1.1, 2.5)	0.025
Day 28±3	25	325.3 (278.2, 380.5)	25	170.5 (112.8, 257.8)	1.9 (1.2, 3.0)	0.005
ChAdOx1						
Day 0	25	43.5 (25.1, 75.4)	25	21.0 (14.5, 30.3)	2.1 (1.1, 3.9)	0.027
Day 10±3	25	111.7 (69.2, 180.4)	25	54.3 (31.2, 94.6)	2.1 (1.0, 4.2)	0.048
Day 28±3	25	134.4 (96.9, 186.4)	25	98.0 (61.0, 157.4)	1.4 (0.8, 2.4)	0.263

* Day 0 antibody titer comparison between heterologous MVC-COV1901 and homologous ChAdOx1 groups: 4-6 weeks interval, 39.0 IU/mL versus 43.5 IU/mL, P = 0.7640; 8-10 weeks interval, 26.6 IU/mL versus 21.0 IU/mL, P = 0.4310.
Abbreviations: GMT, geometric mean titer; CI, confidence interval.
[#] P values were determined by t test (two-tailed).

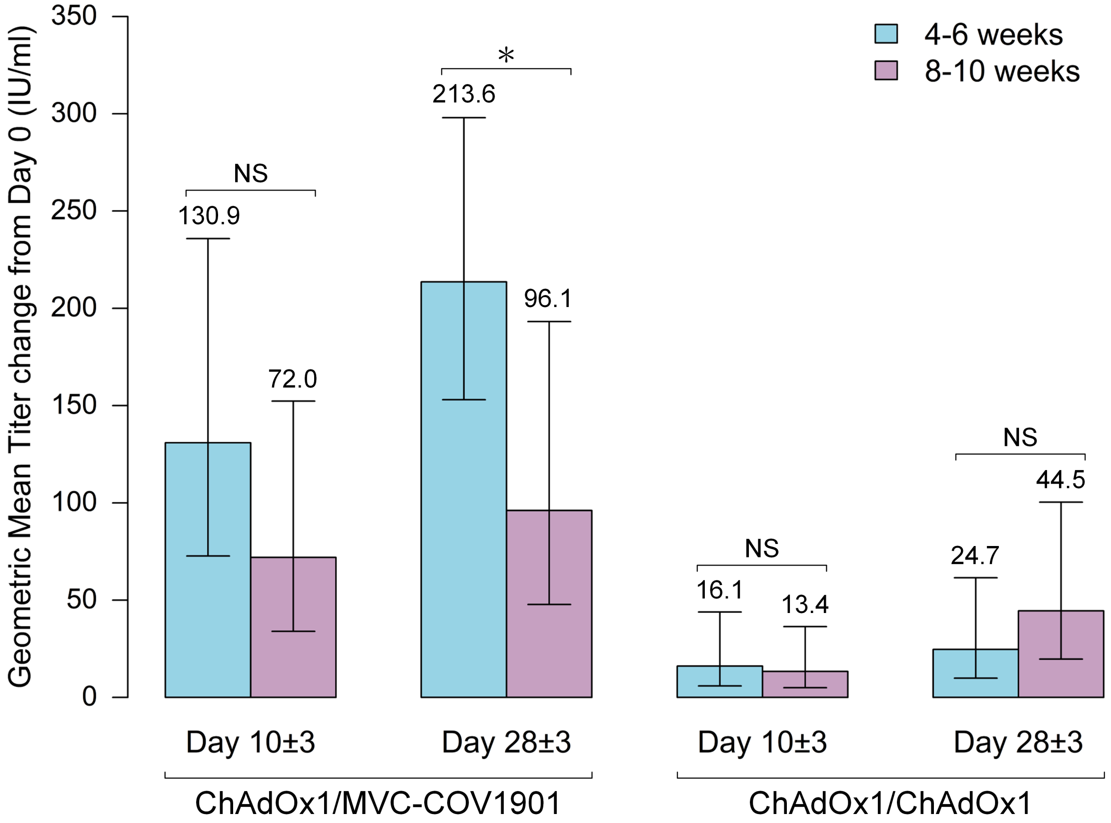

Supplementary Figure 1. Comparisons of changes of antibody titers from baseline of short (4-6 weeks, n = 25) and long (8-10 weeks, n = 25) vaccine dosing intervals. Neutralizing antibody titers were measured by ELISA-based method. Data are presented as geometric mean ± 95% confidence intervals. The significance between two groups was determined using t test (two-tailed). *P < 0.05; NS, not significant. Source data are provided as a Source Data file.

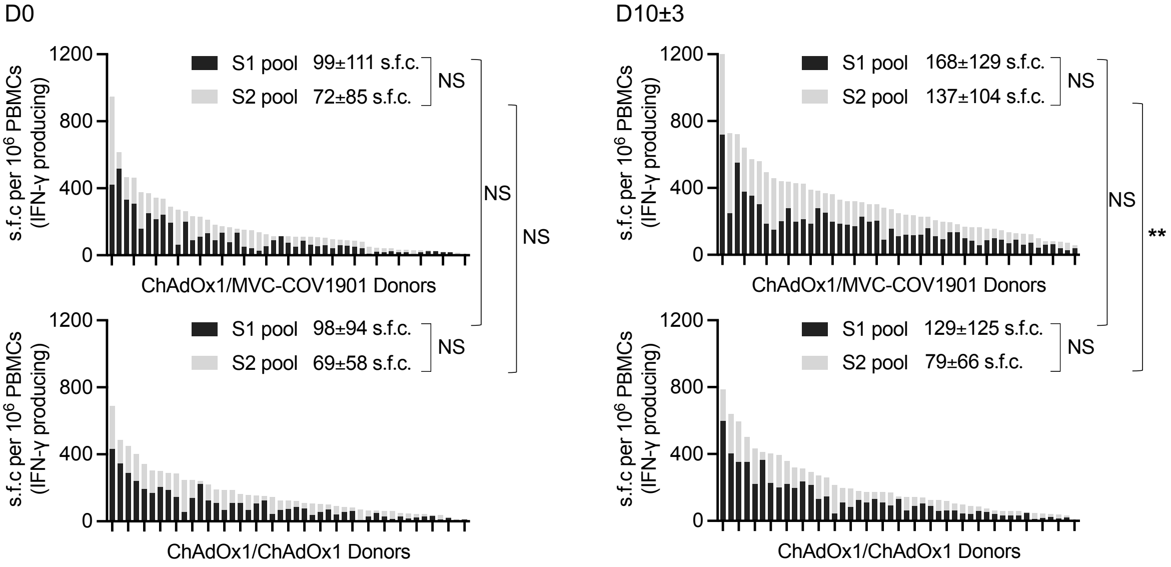

Supplementary Figure 2. Summary data of day 0 and day 10±3 T cell responses to SARS-CoV-2 spike in vaccine recipients (upper panel, ChAdOx1/MVC-COV1901 group, n = 49; lower panel, ChAdOx1/ChAdOx1 group, n=45) according to spike peptide pools. The mean frequency of spike-specific T-cell responses and its standard deviation is shown in the figure. The significance between pools was determined using one-way ANOVA with post hoc Dunn's test. Day 10±3 T cell response, S1 pool comparison, P = 0.0901; S2 pool comparison, P = 0.0070. **P < 0.01; NS, not significant. Source data are provided as a Source Data file.

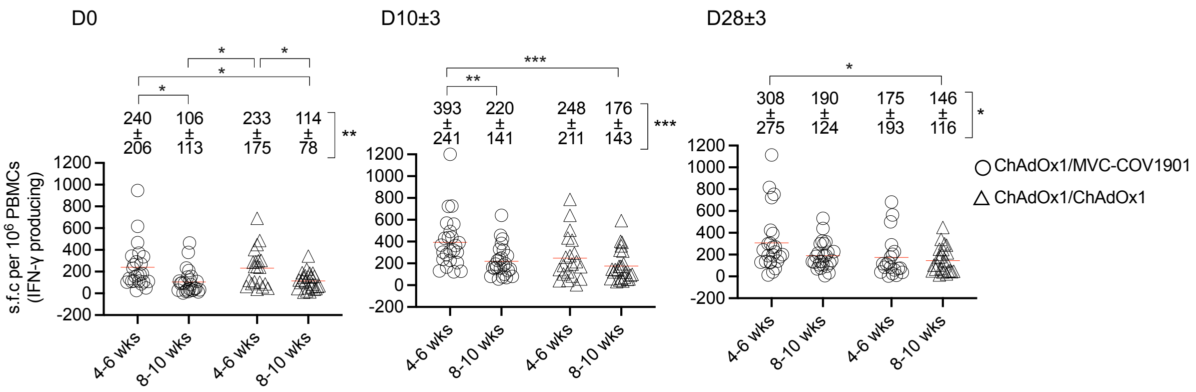

Supplementary Figure 3. Total spike-specific T-cell responses (sum of S1 and S2 subunit responses) prior to, on day 10±3 and day 28±3 after the booster dose in short (4-6 weeks, n = 24 in ChAdOx1/MVC-COV1901 group, n = 20 in ChAdOx1/ChAdOx1 group) and long (8-10 weeks, n = 25 in ChAdOx1/MVC-COV1901 group, n = 25 in ChAdOx1/ChAdOx1 group) vaccine dosing intervals. Each point represents a single recipient and red line represents the mean. The mean frequency of spike-specific T-cell responses and its standard deviation is shown in the figure. The significance was determined using one-way ANOVA with post hoc Dunn's test. Day 0, P = 0.0013, post hoc ChAdOx1/MVC-COV1901 short vs ChAdOx1/MVC-COV1901 long, P = 0.0116; ChAdOx1/MVC-COV1901 short vs ChAdOx1/ChAdOx1 long, P = 0.0206; ChAdOx1/MVC-COV1901 long vs ChAdOx1/ChAdOx1 short, P = 0.0281; ChAdOx1/ChAdOx1 short vs ChAdOx1/ChAdOx1 long, P = 0.0462. Day 10±3, P = 0.0007, post hoc ChAdOx1/MVC-COV1901 short vs ChAdOx1/MVC-COV1901 long, P = 0.0089; ChAdOx1/MVC-COV1901 short vs ChAdOx1/ChAdOx1 long, P = 0.0006. Day 28±3, P = 0.0185, post hoc ChAdOx1/MVC-COV1901 short vs ChAdOx1/ChAdOx1 long, P = 0.0161. *P < 0.05; **P < 0.01; ***P < 0.001. Source data are provided as a Source Data file.

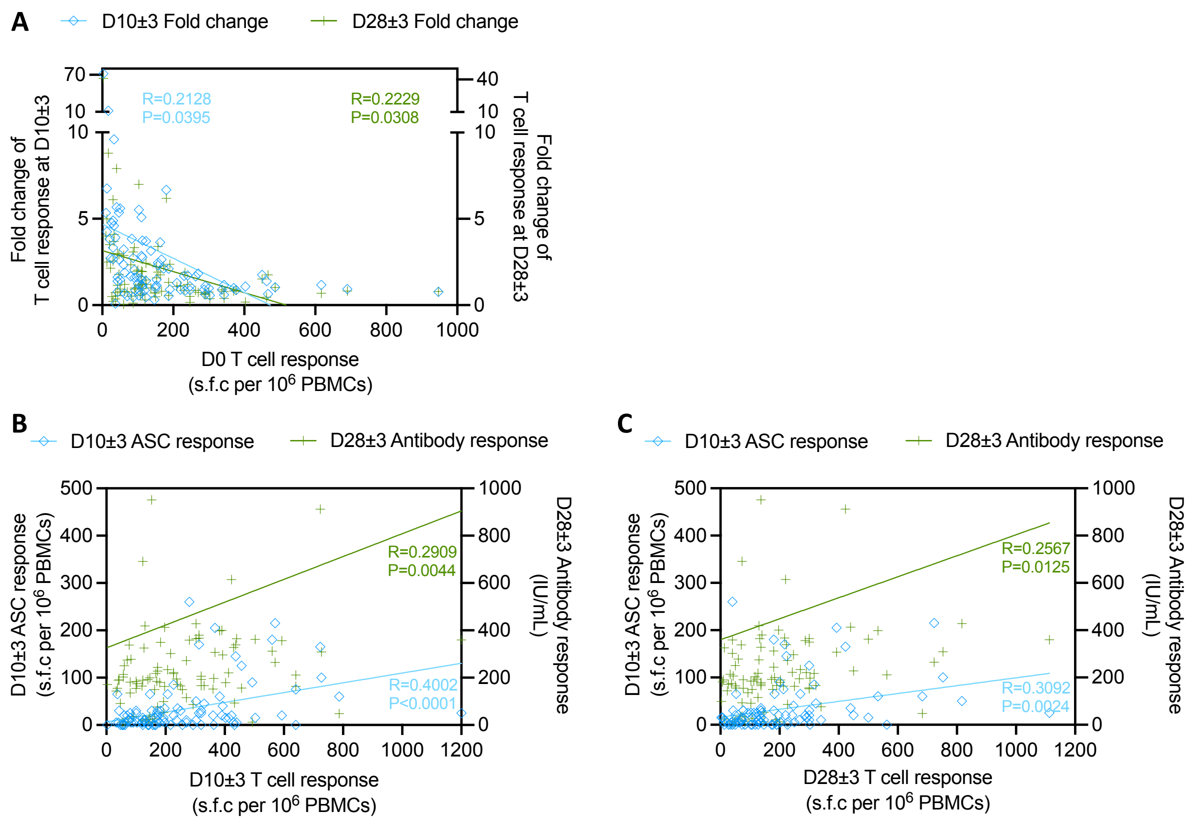

Supplementary Figure 4. (**a**) Relationship between day 0 (baseline) T cell response and the fold change of T cell response after the booster dose (n = 94). Relationship between day 10±3 (**b**) or day 28±3 (**c**) T cell response and ASC and antibody responses (n = 94). The correlation was determined using simple linear regression. Source data are provided as a Source Data file.

Supplementary Note 1. Trial protocol.

CLINICAL STUDY PROTOCOL

A single-blind, randomized study to evaluate the immunogenicity of heterologous prime-boost COVID-19 vaccine schedule

VERSION 1.1

DATES (20210812)

Chih-Jung Chen

Kuan-Ying A. Huang

Lan-Yan Yang

Yhu-Chering Huang

Cheng-Hsun Chiu

Shin-Ru Shih

Chang Gung Memorial Hospital

Table of Contents

Section **Page**

1. Synopsis ***4***

2. Introduction and Rationale ***11***

2.1 Investigational product(s) **13**

2.2 Animal and preclinical study data **13**

2.3 Clinical data **14**

2.4 Risks / benefits Assessment **16**

2.5 Regulatory **20**

3. Objectives and Endpoints ***20***

4. Study Design ***21***

4.1 Overall Design **21**

4.2 Number of Patients **23**

4.3 Schedule of Activities **23**

5. Study Design ***25***

5.1 Inclusion Criteria **25**

5.2 Exclusion Criteria **25**

5.3 Withdrawal criteria **26**

6. Treatments ***27***

6.1. Treatment Administration **27**

6.2 Concomitant Therapy **28**

7. Efficacy Assessments ***28***

8. Safety Assessments ***29***

9. Adverse event reporting ***30***

9.1 Definitions and reports of Adverse Events **30**

9.2 Adverse event follow-up **34**

10. Criteria for the termination of the trial ***35***

11. Statistical Considerations ***35***

11.1 Sample size Determination **35**

11.2 Planned Statistical methods of analysis **36**

11.3 Analysis Population **37**

11.4 Procedure for accounting for missing, unused and spurious data **38**

11.5 Procedures for reporting any deviation(s) from the original statistical plan **38**

12. Direct access to source data/documents ***38***

13. Ethical considerations ***38***

14. Data handling and keeping ***39***

14.1 Data Recording 39
14.2 Record keeping 39
14.3 Source data and Case Report Forms (CRFs) 40
15. Financing and Insurance 40
16. References 41

1. Synopsis

Protocol Title：
A single-blind, randomized study to evaluate the immunogenicity of heterologous prime-boost COVID-19 vaccine schedule

Study Objectives：
To evaluate the immunogenicity of heterologous prime-boost COVID-19 vaccine schedule

Trial Registration：
Taiwan Food and Drug Administration

Sponsor：
Chang Gung Memorial Hospital

Collaborator：
Medigen Vaccine Biologics Corp

Investigational product(s)：

Vaccine	Component	Dose	Route of administration
AstraZeneca COVID-19 vaccine ChAdOx1 nCOV-19 (AZD1222)	a replication-incompetent chimpanzee adenovirus vector that expresses the spike protein	$\geq 2.5 \times 10^{8}$ Inf.U (infectious units) (0.5ml)	Intramuscular
Medigen COVID-19 vaccine (MVC-COV1901)	a subunit vaccine consisting of the prefusion spike protein (S-2P) adjuvanted with CpG 1018 and aluminum hydroxide	15 µg Spike-2P (0.5ml)	Intramuscular

Development Phase: ☐I ■II. ☐III ☐IV 其它_____________ ☐不適用

Study Design：

1. ■ Experimental Group (MVC-COV1901, 1 dose (booster dose), intramuscular)
   ■ Control Group ： □ Placebo
   ■Study Drug (Name、Dose、Usage) _AZD1222, 1 dose (booster dose), intramuscular
   □ Other ______________
2. Blinding：□ Open □ Evaluator-blind ■ Single-blind (patient) □ Double-blind (patient+PI)
   □ Double Dummy □ Other _______________
3. Randomization: ■ Yes □ No
4. ■ Parallel design □ Crossover design □ Other _____________ □ Not applicable
5. Treatment Period： 1 day (days/weeks/months/years) □ Not applicable
6. Study Period: 1 year
6. Dose adjustment：□ Mandatory □ Selectively ■No □ Not applicable
7. Study location：■ Single □ Multi-center □ Global

Endpoints (Outcome measure)：

	Objectives	Outcome measures	Time point
Primary outcome	To determine if the immune response to heterologous prime-boost immunization with ChAdOx1 nCOV-19 (AZD1222) and MVC-COV1901 is non-inferior to homologous prime-boost immunization with ChAdOx1 nCOV-19, in enrolled adult participants	Immunogenicity: Neutralizing antibody against SARS-CoV-2	Day 28 after boost
Secondary outcome	Further determination of immunogenicity to heterologous prime-boost immunization of COVID-19 vaccines	Neutralizing antibody against SARS-CoV-2<br><br>Anti-SARS-CoV-2 Spike antibodies<br><br>Anti-SARS-CoV-2 Nucleocapsid antibodies<br><br>B and T cellular immune responses by ELISpot (only D0, 10, 28)<br><br>Cellular immune response by cytokines (Th1/Th2) (only D0, 10, 28)	D0, 10, 28, 56, 168 after boost
	To evaluate the safety of heterologous & homologous prime-boost immunization of COVID-19 vaccines	Solicited local adverse events (AEs) (up to 7 days after the booster dose of study intervention)<br><br>Solicited systemic AEs (up to 7 days after the booster dose of study intervention)<br><br>Unsolicited AEs (up to 28 days after the	Throughout the study

		booster dose of study intervention)<br><br>AE of special interest (AESI)<br><br>Serious adverse events (SAEs)<br><br>Note:<br>- Solicited local AEs: pain/tenderness, erythema/redness, induration/swelling<br>- Solicited systemic AEs: fever, malaise/fatigue, myalgia, headache, nausea/vomiting, diarrhea	

Inclusion/Exclusion Criteria：

Inclusion criteria

1. Participant is willing and able to give written informed consent for participation in the trial.
2. Male or Female, aged from 20 to 70 years
3. Has received one dose of the AZD1222 within 28-70 days before randomization. Evidence of this will be gathered from medical history and/or medical records including the COVID-19 vaccine registration yellow card.
4. Female participant must:

   a. Be either of non-childbearing potential, i.e. surgically sterilized (defined as having undergone hysterectomy and/or bilateral oophorectomy and/or bilateral salpingectomy; tubal ligation alone is not considered sufficient) or one year post-menopausal;

   b. Or, if of childbearing potential, be abstinent or agree to use medically effective contraception on enrolment continuously until 90 days after boost immunization of study intervention.

   Acceptable forms include:

   i. Implanted hormonal methods of contraception or placement of an intrauterine device or intrauterine system

ii. Established use of hormonal methods (injectable, pill, patch or ring) combined with barrier methods of contraception: condom or occlusive cap (diaphragm or cervical/vault caps) with spermicidal foam/gel/film/cream/suppository

c. Have a negative pregnancy test.

5. In the Investigator's opinion, is able and willing to comply with all trial requirements.

Exclusion criteria

The participant may not enter the trial if ANY of the following apply:

1. Previous receipt of two or more COVID-19 vaccine doses
2. History of anaphylaxis, severe allergic disease or reactions likely to be exacerbated by any component of study vaccines (e.g. hypersensitivity to the active substance or any of the listed ingredients of any study vaccine). This includes latex and polyethylene glycol/macrogol (PEG)
3. Pregnancy, lactation or willingness/intention to become pregnant within 3 months post boost vaccine
4. Malignancy requiring receipt of immunosuppressive chemotherapy or radiotherapy for treatment of solid organ cancer/hematological malignancy within the 6 months prior to enrolment.
5. Bleeding disorder (e.g. factor deficiency, coagulopathy or platelet disorder), or prior history of significant bleeding or bruising following intramuscular injections or venipuncture.
6. Suspected or known current alcohol or drug dependency.
7. Any other significant disease, disorder or finding which may significantly increase the risk to the volunteer because of participation in the study, affect the ability of the volunteer to participate in the study or impair interpretation of the study data.
8. Insufficient level of language to undertake all study requirements in opinion of the Investigators.
9. Known HIV antibody positive.

Study Procedures：

This is a prospective, single blinded randomized homologous/heterologous prime-boost vaccine clinical study, designed to assess the immunogenicity of heterologous prime-boost immunization with AZD1222 and MVC-COV1901 in adults.

Participants will be healthy adults at the age of 20-70 years who have had their first dose of COVID-19 vaccine, AZD1222. All eligible participants of 2 prime-boost interval strata (28 to 42, 56 to 70 days) will be 1:1 randomly assigned to receive a single dose of either:
- Homologous group: Intramuscular injection the same vaccine as their prime dose AZD1222
- Heterologous group: Medigen COVID-19 vaccine MVC-COV1901.

The treatment phase of this study will be conducted in a single-blind fashion such that the subject will not know the identity of the subjects' study treatment assignment. After receiving the treatment, the participants will remain on study for 168 days following the boost vaccination.

For the study primary objective, immunogenicity will be assessed during the duration of the study, including serologic neutralizing antibody titer against SARS-CoV-2, serological quantification of binding antibody to SARS-CoV-2 antigen, SARS-CoV-2 antigen specific B cell and T cell frequencies and cytokine levels.

And Safety will be assessed during the duration of the study as follows:

- Solicited adverse events (AEs; local and systemic) will be assessed for 7 days following each vaccination (Day 0 through Day 7 for the boost vaccination).
- Unsolicited AEs will be recorded for 28 days following the boost vaccination.
- Serious adverse events (SAEs) will be recorded from signing of the informed consent form through Day 168.
- Adverse events of special interest (AESIs) will be recorded from the boost vaccination through Day 168.

This study is going to be conducted in a single medical center in Taiwan. An appropriate number of participants will be screened to achieve approximately 44 evaluable participants for each group. Participants in each group will be divided into two subgroups according to the intervals, 28–42 days and 56–70 days, between the prime and booster doses.

Study group

Group	Prime-Boost (28–42 days and 56–70 days interval)
Heterologous group	1st dose AZD1222, 2nd dose MVC-COV1901
Homologous group (control)	1st dose AZD1222, 2nd dose AZD1222

Flow Chart

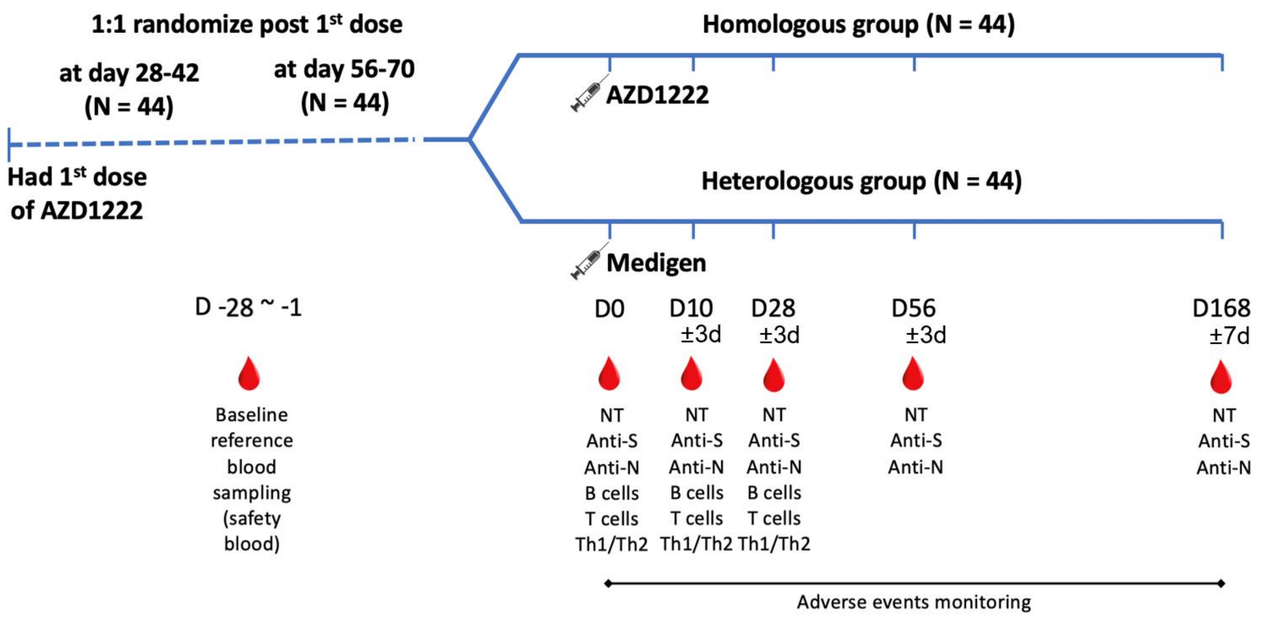

Time-Event scheme

Visit Number	1	2	3	4	5	6	7
Day	**-28 ~ -1**	**0**	**7**	**10 ± 3 days**	**28 ± 3 days**	**56 ± 3 days**	**168 ± 7 days**
Phase	**Screening**	**Boost Vaccination**	**Phone call Follow-up**	**Follow-up**	**Follow-up**	**Follow-up**	**Follow-up**
**Clinic visit**	X	X		X	X	X	X
**Informed consent**	X						
**Inclusion/exclusion criteria**	X						
**Safety bloods**	X			X			
**Medical history**	X						
**Symptom Questionnaire**	X	X					
**Physical examination**	X	X		X	X	X	X
**Height and body weight**	X						
**Pregnancy test (if required)**		X					
**Randomization**		X					
**COVID-19 vaccination**		X					
**Immunogenicity bloods**		X		X	X	X	X
**Diary card review**				X	X		
**Solicited AE**			X				
**Unsolicited AE**		X	X	X	X		
**SAE, AESI**		X	X	X	X	X	X
**Study completion**							X

- Solicited adverse events (AEs; local and systemic) will be assessed for 7 days following each vaccination (Day 1 through Day 7 for the boost vaccination).
- Unsolicited AEs will be recorded for 28 days following the boost vaccination.

Participants will be informed that there may be leftover samples of their blood (after all testing for this study is completed), and that such samples may be stored indefinitely for possible future research that are relevant to vaccine immunogenicity. Participants will be able to decide if they will permit such future use of any leftover samples. With the participants' informed consent, any leftover cells and serum/plasma will be frozen indefinitely for future analysis of COVID-19 and other coronaviruses related diseases or vaccine-related responses. If a participant elects not to permit this, all of that participants' leftover samples will be discarded at the end of the trial. Samples that are to be stored for future research will be transferred to the Chang Gung Memorial hospital Biobank.

1. **Concomitant Treatments**： ■ Not applicable

1. Concomitant Therapy：

2. Prohibited Therapy：

Statistical Methods：

1. Main study Hypothesis：☐ Equality ☐ Superiority ■ Non-inferiority

☐ Equivalence ☐ Other ________

2. Estimated Sample Size：整個試驗預計納入人數 __110__，整個試驗可評估人數 __88__

本中心預計納入人數__110__，本中心可評估人數__88__

3. Efficacy assessment group：☐ Intent-to-treat (ITT) ■ Per-Protocol (**PP)**

☐ Other ________

4. Interim analysis： ■ Yes (blinding analysis of the samples at day 28 following the boost vaccination)
☐No

5. Statistical methods：

This is a phase II single-blinded randomized clinical trial to evaluate the immunogenicity and safety of heterologous prime boost use of AZD1222 and MVC-COV1901 in adults. The primary objective is to determine if the immune response of heterologous group is non-inferior to that observed in homologous group, and the primary endpoints is neutralizing antibody titer CPE NT_{50} at 28 days after second vaccination.

By assuming the non-inferiority margin is 0.67 fold-difference or -0.401 absolute difference of log GMT between heterologous group and homologous group with the standard deviation 0.66, and the true difference of log GMT is 0, the study will need to recruit 44 evaluable participants per group (total 88 participants) to achieve 80% of power at one-sided 2.5% significance level. According to the missing rate 10% and the stratification in 1:1 for prime-boost 28-42 and 56-70 days, we plan to enroll 50 participants for each strata and equally random assigned to each group (25 for Heterologous and 25 for Homologous group) within strata (total 100 participants).

The adjusted mean difference of logGMC will be presented with the two-sided 95% confidence interval (CI). We will claim heterologous boost arm is non-inferior to homologous boost arm if the lower CI lies above -0.401.

For efficacy assessment, descriptive statistics on continuous measurements will include means, medians, standard deviations, and ranges, while categorical data will be summarized using frequency counts and percentages. For the immunogenicity endpoints including immunogenicity, SARS-CoV-2 neutralizing antibody level, serology assessments, and cell-mediated immune responses, the point estimates will be reported with 95% confidence intervals. For the secondary endpoints for comparisons of continuous scale between groups, independent t-test will be considered. Evidence of significant interaction will be assessed at the 5% level.

For safety analysis, the number (%) of subjects with adverse events will be reported. Frequency counts and percentages will also be presented of subjects with serious adverse events, adverse events leading to withdrawal, adverse events by severity and adverse events by relationship to study treatment. Adverse events, clinical laboratory evaluations, and all other safety measures will be analyzed for the Safety population. The estimate will be derived using SAS software [SAS Institute Inc, 1996].

6. Handling of Missing Data：

The level and pattern of the missing data in the baseline variables and outcomes will be reported. The potential causes of any missing data will be investigated and documented as far as possible. Any missing data will be dealt with using methods appropriate to the conjectured missing mechanism and level of missing.

2. Introduction and Rationale

In late 2019, a novel coronavirus emerged and was identified as the cause of a cluster of respiratory infection cases in Wuhan, China. It spread quickly around the world. In March of 2020 a pandemic was declared by the World Health Organization, the virus was formally named as Severe Acute Respiratory Syndrome Coronavirus 2 (SARS-CoV-2) and the resulting disease was named COVID-19. As of 27 May 2020, there have been over 168 million confirmed cases of SARS-CoV-2 infection with over three million deaths (https://covid19.who.int/).

There is no fully effective drug for COVID-19 yet. Nevertheless, several vaccines have been developed and there are four major types of COVID-19 vaccines at the moment, including mRNA-based, vector-based, protein-based, and inactivated vaccines. Representative authorized COVID-19 vaccines are Moderna mRNA-1273 (mRNA-based), Pfizer-BioNTech BNT162b2 (mRNA-based), AZD1222 (vector-based), and Janssen Ad26.COV2.S (vector-based), and Novavax NVX-CoV2373 (protein-based) is under assessment procedure for emergency use in the European Union. Emergency use authorization for several COVID-19 vaccines have been issued in United States (Moderna mRNA-1273, BNT162b2, Ad26.COV2.S) and European Union (AZD1222, Moderna mRNA-1273, BNT162b2, Ad26.COV2.S). These vaccines, i.e., AZD1222 and mRNA-1273, can elicit neutralizing and cellular responses in nonhuman primates without evidence of enhanced disease. They show immunogenicity in human trials and compared anti-SARS-CoV-2 spike antibody and neutralizing antibody titers to convalescent COVID-19 sera are detected. Phase III clinical trials have been conducted for several vaccines, i.e., AZD1222 and mRNA-1273, and the results show variable but generally satisfactory efficacies in preventing symptomatic COVID-19.

In Taiwan, the AZD1222 COVID-19 vaccine has been certified and distributed for use across the country since the end of March 2021. Moderna mRNA-1273 is also available in Taiwan and the vaccine has been certified by Taiwan Food and Drug Administration (TFDA). The adjuvanted protein COVID-19 vaccine developed by Medigen, MVC-COV1901, was officially authorized for emergency use on 19 July 2021 in Taiwan. All these vaccines were developed for use as homologous two-dose regimens. More vaccines using different platforms, the majority of which are expected to be suggested as two dose,

homologous prime/boost schedules, are being studied and evaluated in UK and USA.

Shortages of vaccine supplies are driving Taiwan to slip further behind the rest of the world in the COVID-19 vaccine roll out and approximately 25% of citizens have been administered with COVID-19 vaccine in Taiwan at the end of July 2021. Given the limited supply the authority may prioritize giving the first dose to as many high-risk people as possible in the shortest amount of time. In addition, in view of the anticipated programmatic challenges of immunizing large proportions of the population, there would be advantages to having flexible immunization schedule where the second dose is not necessarily the same as the first dose, i.e. a permissive approach to using heterologous prime-booster schedule.

Accordingly, this study will determine the safety and immunogenicity of heterologous boost schedules for candidate COVID-19 vaccines that are deployed in Taiwan, for which immunogenic data are not known yet. The vaccines to be studied in this protocol will primarily be determined by those made available for population use. Based on the information from the Central Epidemic Command Center (CECC), Taiwan has signed contracts with various sources to purchase nearly 20 million vaccine doses, including 10 million doses of the AstraZeneca vaccine (AZD1222), 5.05 million doses of the Moderna vaccine (mRNA-1273), and 4.76 million doses of unspecified brands through the COVAX program. The AZD1222 vaccine is the primary COVID-19 vaccine available in Taiwan and over 300,000 doses of AZD1222 have been administered as the first dose. We expect the domestic COVID-19 vaccine from Medigen (MVC-COV1901) would be available for use as early as in August 2021 in Taiwan.

Here, we will setup a pilot study to evaluate the safety and immunogenicity of a heterologous prime-boost vaccination strategy with the AZD1222 or mRNA-1273 as the first dose and the MVC-COV1901 as the booster dose. While the WHO and Taiwan CECC have recommended a minimum interval of 8 weeks for AZD1222 nCoV-19. An interval of 4 weeks was approved for the Emergency Use Authorization issued by TFDA for MVC-COV1901. This study will therefore evaluate combinations of vaccines within an 4–10-week window post prime dose. The population to be studied will be adults aged 20-70 years. The reason for this is that this will most likely include the target population for vaccination, as these are the population who are most at risk of severe disease.

2.1 Investigational product(s)

Vaccine	Component	Dose	Route of administration
AstraZeneca COVID-19 vaccine AZD1222 (ChAdOx1 nCOV-19)	a replication-incompetent chimpanzee adenovirus vector that expresses the spike protein	$\geq 2.5 \times 10^8$ Inf.U (infectious units) (0.5ml)	Intramuscular
Medigen COVID-19 vaccine (MVC-COV1901)	a subunit vaccine consisting of the prefusion spike protein (S-2P) adjuvanted with CpG 1018 and aluminum hydroxide	15 µg Spike-2P (0.5ml)	Intramuscular

2.2 Animal and preclinical study data

AstraZeneca COVID-19 vaccine AZD1222 (ChAdOx1 nCOV-19)

AZD1222 is a recombinant replication-defective chimpanzee adenovirus expressing the SARS-CoV-2 Spike surface glycoprotein. Nonclinical studies found AZD1222 to be immunogenic in BALB/c and CD-1 mice, porcine, and NHP models. Based on accumulating nonclinical and clinical data gathered for AZD1222 as well as other SARS-CoV-2 vaccines in development, a 2-dose regimen was selected in order to enhance the immune responses to the virus.

In the non-human primate challenge study, a single administration of AZD1222 significantly reduced viral load in bronchoalveolar lavage fluid and respiratory tract tissue of vaccinated animals as compared to vector controls. Six macaques received a second dose of AZD1222 4 weeks after the first dose. The second dose resulted in increases in both ELISA and neutralizing antibody titers, and fewer areas of the lung contained viral RNA in prime boost group compared to the prime group. In a porcine model, three pigs also received a second dose of AZD1222 4 weeks after the first dose. In the animals that received the booster dose, both antibodies to the SARS-CoV-2 RBD and neutralizing antibodies were boosted after the second dose.

Medigen COVID-19 vaccine MVC-COV1901

This is a subunit vaccine consisting of the prefusion spike protein (S-2P) adjuvanted with CpG

1018 and aluminum hydroxide. Preclinical animal studies in mice showed that S-2P in combination with CpG 1018 and aluminum hydroxide was potently immunogenic, induced high titer of neutralizing antibodies against SARS-CoV-2 and a marked Th1 dominant response. No vaccine-related serious adverse effects were found in the dose-ranging study administered single- or two-dose regimens of S-2P combined CpG 1018 with alum in the animal study.

In the Golden Syrian hamster study, two doses of MVC-COV1901 induced high levels of neutralizing antibodies and an average of 50-fold higher neutralizing titers against SARS-CoV-2 pseudoviruses than vehicle or adjuvant control groups. MVC-COV1901 immunization prevents hamsters from weight loss after SARS-CoV-2 challenge and immunized mice had significantly reduced lung pathology and undetectable lung viral loads compared to unvaccinated animals. The animal data suggest that MCV-COV1901 is highly immunogenic and protective against infection *in vivo*.

2.3 Clinical data

AstraZeneca COVID-19 vaccine AZD1222 (ChAdOx1 nCOV-19)

A phase 1/2, single-blind, randomized controlled trial was conducted in five trial sites in the UK of AZD1222 (ChAdOx1 nCoV-19) expressing the SARS-CoV-2 spike protein compared with a meningococcal conjugate vaccine as control. A total of 1077 participants were enrolled including 544 participants who received at least one dose AZD1222 and 10 participants who received a second dose of AZD1222 4 weeks later. Safety data found the vaccine was generally tolerated, with no treatment-related SAEs reported through 28 days post dose. The most common local solicited AEs were vaccination site pain and tenderness. The most common systemic solicited AEs were chills, feverishness, fever, headache, malaise, and myalgia. The majority of events were mild or moderate in severity and resolved within 1 to 7 days. Following the second dose, a general attenuation in the incidence and severity of local and systemic solicited AEs was observed.

The immunogenicity data from phase 1/2 trial of AZD1222 suggest that a single dose can elicit both humoral and cellular immunogenicity responses and that antibody responses are boosted after a second dose. Spike-specific T-cell responses peaked on Day 14. Anti-Spike IgG responses rose by Day 28, and were boosted 3-fold following a second dose. Neutralising antibody responses against SARS-CoV-2 were detected in 32 (91%) of 35 participants after a single dose when measured in microneutralization (MNA_{80}) and in 35 (100%) participants

when measured in plaque reduction neutralization assay ($PRNT_{50}$). After a booster dose, all participants had neutralising activity (nine of nine in MNA_{80} at day 42 and ten of ten in Marburg VN on day 56).

A multinational phase III randomized trial showed the AZD1222 vaccine had 70.4% efficacy (95% CI 54.8-80.6) in preventing symptomatic COVID-19 at or after 14 days following the second dose. In a subsequent analysis of this trial, vaccine efficacy for symptomatic COVID-19 was 76% from 21 days after receipt of the first dose until receipt of the second dose or day 90, whichever came first, suggesting protection with a single dose. Additionally, receipt of the second dose at 12 weeks or later was associated with higher vaccine efficacy than receipt at <6 weeks (81 versus 55 percent). These findings lend support to extending the time interval for the second dose to 12 weeks. The overall analysis revealed that AZD1222 has an acceptable safety profile and is efficacious against symptomatic COVID-19 in clinical trials.

Medigen COVID-19 vaccine MVC-COV1901

Medigen Vaccine Biologics Corp. (MVC) announced interim analysis result of First-in-Human Trial of COVID-19 vaccine, MVC-COV1901 in a preprint this April (https://www.medrxiv.org/content/10.1101/2021.03.31.21254668v1). In a phase 1, dose-escalation clinical trial of MCV-COV1901 of healthy adults aged 20-49, showed the adjuvanted vaccine containing either 15 μg or 25 μg dose induced neutralizing antibody response against wild type SARS-CoV-2 in all participants. The geometric mean values of serological neutralizing level is 1.8 to 3.9 times those of control convalescent sera. The MCV-COV1901 also induces a Th1-skewed immune response in adult participants. Solicited adverse events were mostly mild and no participants experienced fever. This phase I trial data suggests that the MVC-COV1901 vaccine is safe and elicits remarkable immune responses in 15 μg or 25 μg dose group.

Investigational products, summary of relevant studies

Vaccine	Phase	Trial registration	Route	Dose	Age (yrs)	Number of participants
AstraZeneca COVID-19 vaccine AZD1222 (ChAdOx1 nCOV-19)	Phase 1/2 efficacy, safety and immunogenicity	EudraCT 2020-001072-15	IM	$5x10^{10}$ vp	18-55	1077

AstraZeneca COVID-19 vaccine AZD1222 (ChAdOx1 nCOV-19)	Phase 2/3	EudraCT 2020-001228-32	IM	2.5-5x10^{10} vp	18-64, >65	10,812
AstraZeneca COVID-19 vaccine AZD1222 (ChAdOx1 nCOV-19)	Phase 3	NCT04536051	IM	5x10^{10} vp	>18	10,416
AstraZeneca COVID-19 vaccine AZD1222 (ChAdOx1 nCOV-19)	Adaptive phase 1/2	NCT04444674	IM	5x10^{10} vp	18-65	2130
AstraZeneca COVID-19 vaccine AZD1222 (ChAdOx1 nCOV-19)	Phase 3, double-blind, placebo controlled	NCT04516746	IM	5x10^{10} vp	18-130	32,459
Medigen COVID-19 vaccine MVC-COV1901	Phase 1, prospective, open-labeled	NCT04487210	IM	5, 15, or 25 µg of S-2P	20-49	45
Medigen COVID-19 vaccine MVC-COV1901	Phase 2, double-blind, multicenter	NCT04695652	IM	15 µg of S-2P	>=20	3700

2.4 Risks / benefits Assessment

2.4.1 Unapproved schedules and vaccines

Participants in the homologous group will be receiving a homologous boost with AZD1222, which is with the current approved schedules. Furthermore, those randomized to receive Medigen MVC-COV1901 might be receiving a vaccine that is yet to be approved.

Although the heterologous boost schedule has not been approved, ‘as the vaccine (MCV-COV1901) is based on the spike protein, it is likely the second dose will help to boost the response to the first dose’. Therefore, a person receiving a heterologous boost schedule, is to be

considered as immunized, and to not require additional doses of COVID-19 vaccine, and it is applicable to the MVC-COV1901 vaccine. A growing body of evidence had suggested the heterologous schedule using adenovirus vector vaccine and mRNA vaccine could generate an immune response that surpasses that of the homologous prime/boost schedule in human subjects.

2.4.2 Associated with phlebotomy

Localized bruising and discomfort can occur at the site of venipuncture. Infrequently fainting may occur. These will not be documented as AEs if they occur. The total volume of blood drawn over a nearly 6-month period will be up to 170 ml (+ up to 5 ml per visit if required). This should not compromise these otherwise healthy volunteers, as these volumes are within the limits of 250 mL every 2 months (or 500 mL every 3 months; 1500 mL every year for males, 1000 mL every year for females) for blood donations to the Taiwan Blood Services Foundation. Participants will be asked to refrain from blood donation for the duration of their involvement in the trial.

2.4.3 Allergic reactions

Allergic reactions from mild to severe may occur in response to any constituent of a medicinal product's preparation. Anaphylaxis is known to occur in approximately 1 in 1,000,000 doses of all vaccines but can occur in response to any vaccine or medication.

2.4.4 Behavior change

Participants might feel they can modify their COVID-19 risk behaviors on the assumption that they are protected once vaccinated. Participants will be extensively counselled that they should continue to follow all up to date government advice in relation to COVID-19 precautions during the trial.

2.4.5 Specific risk from vaccine

SAE

There are no SAEs expected for MVC-COV1901. For AZD1222, a rare SAE named thrombosis and thrombocytopenia syndrome (TTS) had been reported to occur in approximately 10 cases per million doses of vaccination in Europe. The incidence of TTS was higher in young adults and most of the cases occurred in women at the age range of 21 – 77 years within 4 – 28 days post the first vaccination dose. The incidence was estimated to be 2 cases per million doses after the 2nd vaccine dose. The thrombosis usually occurred in cerebral

veins, splanchnic veins, and the veins in lower limbs or lungs. A low platelet count of 7 –113,00 x 10^9/L in peripheral blood and presence of the autoantibody anti-platelet factor 4 were common laboratory finding. The frequently mentioned symptoms included persistent headache, backache and abdominal pain, and visual disturbance and leg/arm weakness. High dose immunoglobulin intravenous, steroid and plasmapheresis were reported to be effective in the management of patients with TTS.

The foreseeable ARs following vaccination are as follows:

**Local reactions** (injection site common and expected)	**Systemic reactions** (common and expected)(mild to moderate)	**Systemic reactions** (uncommon and expected)(mild to moderate)	**Laboratory events**
Tenderness	Fatigue	Abdominal pain	Transient neutropenia from baseline is common and expected
Pain	Headache	Feeling dizzy	
Warmth	Myalgia	Decreased appetite	
Redness	Arthralgia	Enlarged lymph nodes	
Itching	Nausea or vomiting	Excessive sweating, itching skin or rash	
Swelling or bruising at the injection site	Malaise		
Lump at the injection site	Chills		
	Feverishness		
	Fever		
	Coryza, sore throat, runny nose		

2.4.6 Receiving a vaccine schedule that is not found to be non-inferior

It is possible that the combinations of vaccine schedule used in this trial is, on analysis found not to be non-inferior to the comparative homologous schedule. This is the reason the trial is being performed. This will be mitigated by the possibility of further vaccination, as advised by the oversight committees. If a study group was found to have an antibody response that did not meet the non-inferiority criteria, they would be offered a booster dose of vaccine as advised by the DSMB.

2.4.7 Antibody-dependent enhancement and immunopathology

Safety concerns around the use of some viral antigens as a vaccine antigen have been raised following historical and limited reports of immunopathology and antibody dependent enhancement reported in vitro and post SARS-CoV challenge in mice, ferrets and non-human primates immunized with whole SARS-CoV inactivated or full-length S-protein-based vaccines, including a study using Modified Vaccinia Ankara as a vector. To date, there has been one report of lung immunopathology following MERS-CoV challenge in mice immunized with an inactivated MERS-CoV candidate vaccine. In preclinical studies of ChAdOx1 immunization and MERS-CoV challenge, no ADE was observed in hDPP4 transgenic mice, dromedary camels or non-human primates. The possibility of ADE has also been evaluated in clinical and pre-clinical studies of the vaccines used in this trial. Nevertheless, this risk will not have been assessed for heterologous boost schedules. Participants will be made aware of this theoretical risk.

2.4.8 Emerging Thrombosis with Thrombocytopenia Association with vaccination

There have extremely rare reports of cerebral venous sinus thrombosis (and thrombosis of other major veins) with concurrent thrombocytopenia that have occurred after AZD1222 vaccination. Previously, the Taiwan's CECC issued a warning of unconfirmed risks of an increased rate of blood clots associated with the vaccine and based on that warning, the vaccines were not recommended for people taking oral contraceptives or undergoing hormone therapy. Nevertheless, the CECC has withdrawn its recommendation that people taking oral contraceptives or undergoing hormone therapy not take the AstraZeneca AZD1222 vaccine, based on updated research findings, on May 21st.

All participants in this study will be provided with up-to-date information from regulators on

this finding via the participant information sheet. They will also be provided with other relevant documentation from regulators and/or public health authorities related to this association and possible risks of vaccination that is also being provided in the investigation site. Participants who will potentially receive the AZD1222 (ChAdOx1 nCOV-19) will be given public health documents specific to this vaccine. Participants will be advised to be aware of possible signs and symptoms of blood clots and to have a low threshold to contact trial teams if experiencing these or other symptoms.

2.5 Regulatory

This study will be conducted in compliance with the protocol approved by the Institutional Review Board, and according to Good Clinical Practice standards. No deviation from the protocol will be implemented without the prior review and approval of the IRB except where it may be necessary to eliminate an immediate hazard to a research subject. In such case, the deviation will be reported to the IRB as soon as possible.

3. Objectives and Endpoints

	Objectives	Outcome measures	Time point
Primary outcome	To determine if the immune response to heterologous prime-boost immunization of COVID-19 vaccines is non-inferior to that observed after homologous prime-boost immunization of COVID-19 vaccines, in enrolled adult participants	Immunogenicity: Neutralizing antibody against SARS-CoV-2	Day 28 after boost
Secondary outcome	Further determination of immunogenicity to heterologous prime-boost immunization of COVID-19 vaccines	Neutralizing antibody against SARS-CoV-2<br><br>Anti-SARS-CoV-2 Spike antibodies<br><br>Anti-SARS-CoV-2 Nucleocapsid antibodies<br><br>B and T cellular immune responses by ELISpot (only D0, 10, 28)<br><br>Cellular immune response by cytokines	D0, 10, 28, 56, 168 after boost

		(Th1/Th2) (only D0, 10, 28)	
	To evaluate the safety of heterologous & homologous prime-boost immunization of COVID-19 vaccines	Solicited local adverse events (AEs) (up to 7 days after the boost dose of study intervention)<br><br>Solicited systemic AEs (up to 7 days after the boost dose of study intervention)<br><br>Unsolicited AEs (up to 28 days after the boost dose of study intervention)<br><br>AE of special interest (AESI)<br><br>Serious adverse events (SAEs)<br><br>Note:<br>- Solicited local AEs: pain/tenderness, erythema/redness, induration/swelling<br>- Solicited systemic AEs: fever, malaise/fatigue, myalgia, headache, nausea/vomiting, diarrhea	Throughout the study

4. Study Design

4.1 Overall Design

A single-blind, randomized, pilot study to evaluate the safety and immunogenicity of heterologous prime-boost COVID-19 vaccine schedule.

4.1.1 Setting

Single-center study conducted through academic and clinical trials sites.

4.1.2 Trial duration

Total duration of each participant will be 6 months from the enrolment. The total trial period will be approximately 1 year

4.1.3 Study groups

A total of 100 (+ up to an additional 10%) participants will be enrolled, all of whom will have had their first dose of AZD1222 vaccine under the government-funded COVID-19 vaccination program. Participants stratified by two prime-boost intervals (28-42 and 56-70 days) will be randomized equally to receive either a homologous or heterologous boost (50 per group) as outlined below.

Group	Arm	Prime-Boost (N=50) (28-42, days interval)	Prime-Boost (N=50) (56-70 days interval)	Visits
Experimental group	Heterologous schedule	1st dose AZD1222<br>2nd dose MVC-COV1901<br>(N=25)	1st dose AZD1222<br>2nd dose MVC-COV1901<br>(N=25)	Day -28~-1, 0, 7, 10, 28, 56, 168
Control group	Homologous schedule	1st dose AZD1222<br>2nd dose AZD1222<br>(N=25)	1st dose AZD1222<br>2nd dose AZD1222<br>(N=25)	Day -28~-1, 0, 7, 10, 28, 56, 168

Both groups will have visits: -28~-1, 0, 7, 10, 28, 56, 168

The study will be single-blind, i.e., while staff involved in study delivery will be aware of what vaccine schedule the participant is receiving, the participant themselves will remain blinded to their booster vaccine. This blind will be maintained by applying a masking tape over the vaccine syringe. Laboratory staff will also be blinded to the vaccine schedule received.

Participants who acquire new infection with SARS-CoV-2 will have an additional study visit for clinical assessment, to take blood tests for immunological assessment and to take a sample for isolation of virus. They may also have nasal fluid and saliva samples taken.

On 10th February 2021 the WHO issued revised recommendations that the Oxford/AstraZeneca AZD1222 (ChAdOx1-nCoV-19) vaccine be given at an 8–12-week boost interval (World Health Organization 2021). The boost interval for both vaccine schedule combinations will be 56 days, this will therefore be in keeping with the WHO recommendations for the homologous AZD1222 schedule, and will maintain blinding.

Flow Chart (試驗流程圖)：

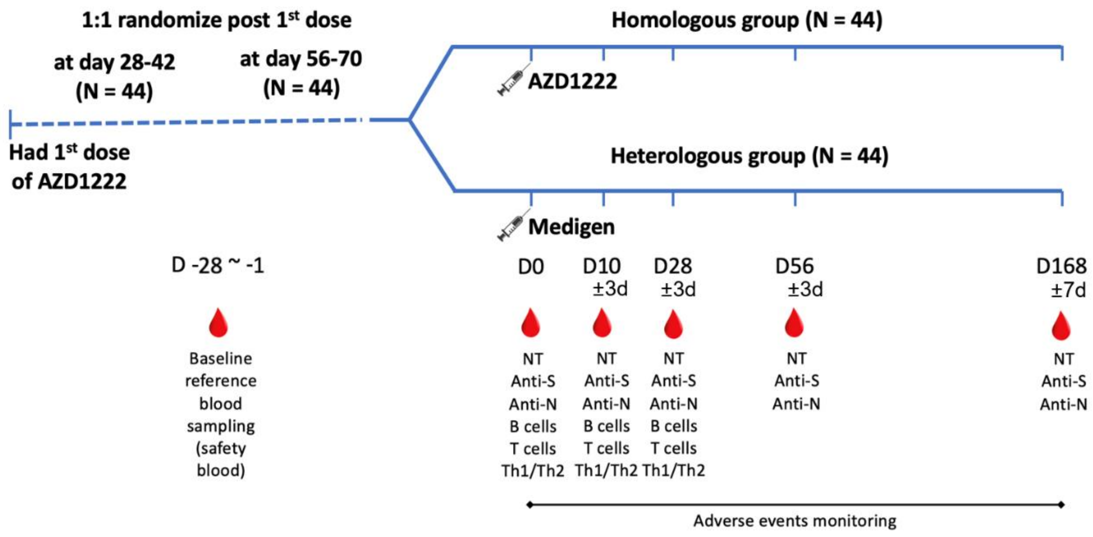

4.2 Number of Patients

A total of 100 (+ up to an additional 10%) participants will be enrolled, all of whom will have had their first dose of COVID-19 vaccine under the government-funded COVID-19 vaccination program. Participants of 2 prime-boost interval strata will be randomized to receive either a homologous or heterologous boost (50 per group).

4.3 Schedule of Activities

Time-Event scheme:

Visit Number	1	2	3	4	5	6	7
Day	-28~-1	0	7	10<br>± 3 days	28<br>± 3 days	56<br>± 3 days	168<br>± 7 days
Phase	Screening	Boost Vaccination	Phone call Follow-up	Follow-up	Follow-up	Follow-up	Follow-up
**Clinic visit**	X	X		X	X	X	X
**Informed consent**	X						
**Inclusion/exclusion criteria**	X						
**Randomization**		X					
**Safety bloods**	X			X			
**Medical history**	X						
**Symptom Questionnaire**	X	X					
**Physical examination**	X	X		X	X	X	X
**Height and body weight**	X						
**Pregnancy test (if required)**		X					
**COVID-19 vaccination**		X					
**Immunogenicity bloods**		X		X	X	X	X
**Diary card review**			X	X	X		
**Solicited AE**			X				
**Unsolicited AE**			X	X	X		
**SAE, AESI**			X	X	X	X	X
**Study completion**							X

Blood sampling:

Study timeline	Screening	0	10 ± 3 days	28 ± 3 days	56 ± 3 days	168 ± 7 days
**Safety bloods**	1 x full blood count (up to 2ml)<br><br>1 x Biochem (up to 3 ml)		1 x full blood count (up to 2ml)<br>1 x Biochem (up to 3 ml)			
**COVID-19 vaccination**		X				
**Primary endpoint**				Neutralizing antibody		
**Secondary endpoint**		Neutralizing antibody; Anti-Spike antibodies; Anti-Nucleocapsid antibodies; B and T cellular immune responses by ELISpot*; Cellular immune response by cytokines (Th1/Th2)	Neutralizing antibody; Anti-Spike antibodies; Anti-Nucleocapsid antibodies; B and T cellular immune responses by ELISpot*; Cellular immune response by cytokines (Th1/Th2)	Neutralizing antibody; Anti-Spike antibodies; Anti-Nucleocapsid antibodies; B and T cellular immune responses by ELISpot*; Cellular immune response by cytokines (Th1/Th2)	Neutralizing antibody; Anti-Spike antibodies; Anti-Nucleocapsid antibodies	Neutralizing antibody; Anti-Spike antibodies; Anti-Nucleocapsid antibodies
**No of blood sampling and tubes**	1 x full blood count (up to 2ml)<br><br>1 x Biochem (up to 3 ml)	Up to 20 ml serum tube<br><br>*Up to 20 mL Li Heparin or equivalent tubes	Up to 20 ml serum tube<br><br>*Up to 20 mL Li Heparin or equivalent tubes<br><br>1 x full blood count (up to 2ml)<br><br>1 x Biochem (up to 3 ml)	Up to 20 ml serum tube<br><br>*Up to 20 mL Li Heparin or equivalent tubes	Up to 20 ml serum tube	Up to 20 ml serum tube
**Total volume per visit**	Up to 5 mL	Up to 40 mL	Up to 45 mL	Up to 40 mL	Up to 20 mL	Up to 20 mL
**Total volume by end of study**	Up to 170 mL<br>At any point in the study, a repeat of safety bloods may be recommended at the clinical discretion of the investigator. This will result in up to an extra 5 ml per repeat blood sample.					

5. Study Design

5.1 Inclusion Criteria

1. Participant is willing and able to give written informed consent for participation in the trial.
2. Male or Female, aged from 20 to 70
3. Has received one dose of the AZD1222 within 28~70 days before randomization. Evidence of this will be gathered from medical history and/or medical records.
4. Female participant must:

   a. Be either of non-childbearing potential, i.e. surgically sterilized (defined as having undergone hysterectomy and/or bilateral oophorectomy and/or bilateral salpingectomy; tubal ligation alone is not considered sufficient) or one year post-menopausal;

   b. Or, if of childbearing potential, be abstinent or agree to use medically effective contraception on enrolment continuously until 90 days after boost immunization of study intervention.

   Acceptable forms include:

   i. Implanted hormonal methods of contraception or placement of an intrauterine device or intrauterine system

   ii. Established use of hormonal methods (injectable, pill, patch or ring) combined with barrier methods of contraception: condom or occlusive cap (diaphragm or cervical/vault caps) with spermicidal foam/gel/film/cream/suppository

   c. Have a negative pregnancy test.
5. In the Investigator's opinion, is able and willing to comply with all trial requirements.

5.2 Exclusion Criteria

The participant may not enter the trial if ANY of the following apply:

1. Previous receipt of two or more COVID-19 vaccine doses
2. History of anaphylaxis, allergic disease or reactions likely to be exacerbated by any component of study vaccines (e.g. hypersensitivity to the active substance or any of the listed ingredients of any study vaccine). This includes latex and polyethylene glycol/macrogol (PEG)

3. Pregnancy, lactation or willingness/intention to become pregnant within 3 months post boost vaccine
4. Malignancy requiring receipt of immunosuppressive chemotherapy or radiotherapy for treatment of solid organ cancer/hematological malignancy within the 6 months prior to enrolment.
5. Bleeding disorder (e.g. factor deficiency, coagulopathy or platelet disorder), or prior history of significant bleeding or bruising following intramuscular injections or venipuncture.
6. Suspected or known current alcohol or drug dependency.
7. Any other significant disease, disorder or finding which may significantly increase the risk to the volunteer because of participation in the study, affect the ability of the volunteer to participate in the study or impair interpretation of the study data.
8. Insufficient level of language to undertake all study requirements in opinion of the Investigators.
9. Known HIV antibody positive.

5.3 Withdrawal criteria

The Investigator may withdraw the participant at any time in the interests of the participants' health and well-being. In addition, the participant may withdraw/be withdrawn for any of the following reasons:

1. Administrative decision by the Investigator
2. Ineligibility (either arising during the study or retrospectively, having been overlooked at screening).
3. Significant protocol deviation
4. Participant non-compliance with study requirements
5. An AE, which requires discontinuation of the study involvement or results in inability to continue to comply with study procedures

The reason for withdrawal will be recorded. If withdrawal is due to an AE, appropriate follow-up visits or medical care will be arranged, with the agreement of the volunteer, until the AE has resolved, stabilized or a non-trial related causality has been assigned.

If the participant chooses to withdraw after receipt of a vaccine, they will not be unblinded as this

will not change clinical action for them. If a participant withdraws from the study, storage of samples will continue unless the participant specifically requests otherwise. Any data collected before their withdrawal will still be used in the analysis for safety and trial integrity; if the participant requests this could be de-identified following the end of the study.

In cases of participant withdrawal, long-term safety data collection, including some procedures such as safety bloods, may continue as appropriate if participants have received any vaccine doses through the trial, unless they decline any further follow-up.

6. Treatments

6.1. Treatment Administration

6.1.1 AstraZeneca COVID-19 vaccine AZD1222 (ChAdOx1 nCOV-19)

AZD1222 (ChAdOx1 nCoV-19) is a recombinant replication-defective chimpanzee adenovirus expressing the SARS-CoV-2 spike (S) surface glycoprotein with a leading tissue plasminogen activator signal sequence. S is a type I, trimeric, transmembrane protein located at the surface of the viral envelope, giving rise to spike shaped protrusions from the virion. The S proteins subunits are responsible for cellular receptor ACE-2 binding via the receptor-binding domain and fusion of virus and cell membranes, thereby mediating the entry of SARS-CoV-2 into the target cells. The S protein has an essential role in virus entry and determines tissue and cell tropism, as well as host range.

AZD1222 expresses a codon-optimized coding sequence for Spike protein from the SARS-CoV-2 genome sequence accession MN908947. ChAd is a non-enveloped virus, and the glycoprotein antigen is not present in the vector but is only expressed once the genetic code within the vector enters the target cells. The vector genes are also modified to render the virus replication incompetent, and to enhance immunogenicity. Once the vector is in the nucleus, mRNA encoding the spike protein is produced that then enters the cytoplasm. This then leads to translation of the target protein which act as an intracellular antigen.

Dosage, scheduling and packaging

The dose of AstraZeneca COVID-19 vaccine is 0.5ml. The vaccine should be administered intramuscularly. The schedule will be one dose, at 56 days from prime COVID-19 vaccine dose. The AstraZeneca vaccine is supplied in packs of 10 vials. Each vial contains 8 to 10 doses of vaccine, and is a colorless to slightly yellow, clear to slightly opaque liquid. Each dose is

prepared by withdrawing 0.5 mL from a vial in a sterile 1 mL or equivalent syringe.

6.1.2 Medigen COVID-19 vaccine MVC-COV1901

MVC-COV1901 contains prefusion-stabilized SARS-CoV-2 spike protein, S-2P, adjuvanted with CpG 1018 and aluminum hydroxide. The S-2P form was created by mutation of the S1/S2 furin-recognition site 682-RRAR-685 to GSAS to produce a single-chain S0 protein, and the 986-kV-987 was mutated to PP.

Dosage, scheduling and packaging

Each MVC-COV1901 vaccine contained 15 μg of S-2P, administered as a single 0.5 mL intramuscular injection. The vaccine was produced in the Medigen Vaccine Biologics Corporation facility which is compliant with the current good manufacturing practices (cGMP).

6.2 Concomitant Therapy

Concomitant medications taken at enrolment will be recorded, as will new medications taken within the 28 days each immunization. Subsequently only new medications taken in response to a medically attended adverse event up until 3 months post boost will be recorded.

7. Efficacy Assessments

Immunogenicity Assessments

Serum samples for immunogenicity assessments will be collected according to the protocol. Samples will be collected, labelled, stored, and shipped as detailed in the Laboratory Manual.

SARS-CoV-2 Neutralizing Antibody Assessments

Serum samples to measure SARS-CoV-2 neutralizing antibody levels will be collected from participants according to the timepoints specified in the protocol. Authorized laboratories will measure neutralizing antibodies to SARS-CoV-2 using validated wild-type neutralization assay in the Vero E6 cell model. The neutralizing titer will be defined as the reciprocal of the highest dilution capable of inhibiting 50% of cytopathic effect (CPE NT50), which was calculated in using the Reed-Muench method. The reference serum will be included in the assay as control.

The immunogenicity endpoints will comprise the geometric mean titer of wild type virus neutralizing antibody titers. The geometric mean titer will be presented with two-sided 95% CI.

SARS-CoV-2 Serology Assessments

Serum samples will be collected to assess SARS-CoV-2 antigen-specific antibody levels from all participants according to the protocol. Authorized laboratories will assess serologic responses to Spike and Nucleocapsid antigens and serologic assessment will also be assessed quantitatively using a validated immunoassay.

Assessment of Cell-mediated Immune Responses

Cell-mediated immune responses (ie, B-cell and T-cell responses) will be assessed by characterizing PBMCs using methods that may include B-cell and T-cell ELISpot assays to SARS-CoV-2 antigens, flow cytometry after intracellular cytokine staining, and other methodology as determined by the Sponsor and/or authorized laboratories. Data on Th1/Th2 polarization after boost vaccination will be provided.

8. Safety Assessments

These will be processed at agreed laboratories and destroyed in accordance with standard processes. They will include:

1. Haematology – Full Blood Count

2. Biochemistry – Sodium, Potassium, Creatinine, Albumin, Liver Function Tests (ALT, AST, Total Bilirubin) and if relevant C-reactive protein (CRP)

Toxicity grading scale for Laboratory AEs

		Units	Lab range	Grade 1	Grade 2	Grade 3	Grade 4
**Hematology**							
Hemoglobin	Low	g/dL	Male :13.5~17.5<br>Female :12~16	<LLN - 10.0 g/dL	Hgb <10.0 - 8.0 g/dL	Hgb <8.0 g/dL	Life-threatening consequences; urgent intervention indicated
White Blood Cells	Low	1000/uL	Male3.9~10.6<br>Female 3.5~11	<LLN - 3000/mm3	<3000 - 2000/mm3	<2000 - 1000/mm3	<1000/mm3
Platelets	Low	1000/uL	150-400	<LLN - 75.0 x 10e9 /L	<75.0 - 50.0 x 10e9 /L	<50.0 - 25.0 x 10e9 /L	<25.0 x 10e9 /L
Neutrophils	Low	%	42~74	<LLN - 1500/mm3	<1500 - 1000/mm3;	<1000 - 500/mm3	<500/mm3
Lymphocytes	Low	%	20~56	<LLN - 800/mm3	<800 - 500/mm3	<500 - 200/mm3	<200/mm3
Eosinophils	Elevated	%	0-5	>ULN and >Baseline		Steroids initiated	
**Biochemistry**							
Sodium	Elevated	mmol/L	146	>ULN - 150 mmol/L	>150 - 155 mmol/L; intervention initiated	>155 - 160 mmol/L; hospitalizati on indicated	>160 mmol/L; life-threatening consequences
Sodium	Low	mmol/L	136	<LLN - 130 mmol/L	125-129 mmol/L and	120-124 mmol/L	<120 mmol/L; life-threatening

					asymptomatic	regardless of symptoms	consequences
Potassium	Elevated	mmol/L	5.1	>ULN - 5.5 mmol/L	>5.5 - 6.0 mmol/L	>6.0 - 7.0 mmol/L	>7.0 mmol/L
Potassium	Low	mmol/L	3.5	<LLN - 3 g/d	<3 - 2 g/d	<2 g/d	Life-threatening consequences; urgent intervention indicated
Creatinine	Elevated	mg/dL	0.3-1.2	>ULN - 1.5 x ULN	>1.5 - 3.0 x ULN	>3.0 - 6.0 x ULN	>6.0 x ULN
Total Bilirubin	Elevated	mg/dL	0.3~1.2	>ULN - 1.5 x ULN	>1.5 - 3.0 x ULN	>3.0 - 10.0 x ULN	>10.0 x ULN
ALT	Elevated	IU/L	0-36	>ULN - 3.0 x ULN	>3.0 - 5.0 x ULN	>5.0 - 20.0 x ULN	>20.0 x ULN
AST	Elevated	IU/L	0-34	>ULN - 3.0x	>3.0 - 5.0 x ULN	>5.0 - 20.0 x ULN	>20.0 x ULN
Albumin	Low	g/dL	3.5~5.4	<LLN - 3 g/dL	<3 - 2 g/dL	<2 g/dL	Life-threatening consequences; urgent intervention indicated -
CRP	Elevated	mg/L	<5				

Normal ranges may vary between sites and gradings may be adapted between sites

9. Adverse event reporting

9.1 Definitions and reports of Adverse Events

9.1.1 Safety reporting window

Safety reporting for the trial will commence once the first participant is consented; and will end 10 months after the last participant has received the boost dose of an IMP for SAEs and Adverse Events of Special Interest (AESI)s.

For individual participants the reporting period begins when they are consented, and ends 10 months after the boost dose of vaccine for SAE’s and AESI’s.

Principal Investigator will report SAEs to the IRB of Chang Gung Medical Foundation according to the Serious Adverse Event Reporting Procedures and Guidelines as posted in the Clinical Trials Resource on the website of Chang Gung Medical Foundation IRB. SAE reports to the IRB should include the following information when calling the Medical Monitor:

- Date and time of the SAE
- Date and time of the SAE report
- Name of reporter
- Call back phone number
- Affiliation/Institution conducting the study
- Protocol number

- Title of protocol.
- Description of the SAE, including attribution to drug and expectedness

9.1.2 Adverse Event Definitions

**Adverse Event (AE)**	Any untoward medical occurrence in a participant to whom a medicinal product has been administered, including occurrences which are not necessarily caused by or related to that product.
**Adverse Reaction (AR)**	An untoward and unintended response in a participant to an investigational medicinal product which is related to any dose administered to that participant.<br>The phrase "response to an investigational medicinal product" means that a causal relationship between a trial medication and an AE is at least a reasonable possibility, i.e. the relationship cannot be ruled out.<br>All cases judged by either the reporting medically qualified professional or the Sponsor as having a reasonable suspected causal relationship to the trial medication qualify as adverse reactions.
**Adverse Events of Special Interest (AESI)**	Adverse events identified as being of particular relevance to the IMP's. These will also reported as an SAE, if meeting SAE criteria (e.g. hospitalization)
**Serious Adverse Event (SAE)**	A serious adverse event is any untoward medical occurrence that:<br>- Results in death<br>- Is life-threatening<br>- Requires inpatient hospitalization or prolongation of existing hospitalization<br>- Results in persistent or significant disability/incapacity<br>- Consists of a congenital anomaly or birth defect*<br><br>Other 'important medical events' may also be considered a serious adverse event when, based upon appropriate medical judgement, the event may jeopardize the participant and may require medical or surgical intervention to prevent one of the outcomes listed above.<br>NOTE: The term "life-threatening" in the definition of "serious" refers to an event in which the participant was at risk of death at the time of the event; it does not refer to an event which hypothetically might have caused death if it were more severe.
**Serious Adverse Reaction (SAR)**	An adverse event that is both serious and, in the opinion of the reporting Investigator, believed with reasonable probability to be due to one of the trial treatments, based on the information provided.
**Suspected Unexpected Serious Adverse Reaction (SUSAR)**	A serious adverse reaction, the nature and severity of which is not consistent with the Reference Safety Information for the medicinal product in question set out:<br>- In the case of a product with a marketing authorization, in the approved summary of product characteristics (SmPC) for that product<br>- In the case of any other investigational medicinal product, in the approved investigator's brochure (IB) relating to the trial in question

NB: to avoid confusion or misunderstanding of the difference between the terms "serious" and "severe", the following note of clarification is provided: "Severe" is often used to describe intensity of a specific event, which may be of relatively minor medical significance.

"Seriousness" is the regulatory definition supplied above.

The severity of clinical and laboratory adverse events will be assessed according to scales based on FDA toxicity grading scales for healthy adult volunteers enrolled in preventive vaccine clinical trials, listed in the Clinical Study Plan and the Tables below.

Severity grading for local adverse events

Adverse Event	Grade	Intensity
**Pain at injection site**	1	Pain that is easily tolerated
	2	Pain that interferes with daily activity
	3	Pain that prevents daily activity
	4	A&E visit or hospitalization
**Tenderness**	1	Mild discomfort to touch
	2	Discomfort with movement
	3	Significant discomfort at rest
	4	A&E visit or hospitalization
**Erythema at injection site***	1	2.5 - 5 cm
	2	5.1 - 10 cm
	3	>10 cm
	4	Necrosis or exfoliative dermatitis
**Induration/Swelling at injection site**	1	2.5 – 5 cm and does not interfere with activity
	2	5.1 - 10 cm or interferes with activity
	3	>10 cm or prevents daily activity
	4	Necrosis
***erythema ≤2.5cm is an expected consequence of skin puncture and will therefore not be considered an adverse event**		

Severity grading criteria for physical observations.

Vital Signs	Grade 1 (mild)	Grade 2 (moderate)	Grade 3 (severe)	Grade 4 Potentially Life threatening
**Fever (Oral - °C)**	38.0 - 38.4	38.5 – 38.9	39.0 - 40	> 40
**Tachycardia (bpm)***	101 - 115	116 – 130	>130	A&E visit or hospitalization for arrhythmia
**Bradycardia (bpm)****	50 – 54	45 – 49	<45	A&E visit or hospitalization for arrhythmia
**Systolic hypertension (mmHg)**	141 - 150	151 – 155	≥155	A&E visit or hospitalization for malignant hypertension
**Diastolic hypertension (mmHg)**	91 - 95	96 – 100	>100	A&E visit or hospitalization for malignant hypertension
**Systolic hypotension (mmHg)*****	85 - 89	80 – 84	<80	A&E visit or hospitalization for hypotensive shock
**Respiratory Rate (breaths per minute)**	17 - 20	21-25	>25	Intubation
***Taken after ≥10 minutes at rest **When resting heart rate is between 60 – 100 beats per minute. Use clinical judgement when characterizing bradycardia among some healthy subject populations, for example, conditioned athletes. ***Only if symptomatic (e.g. dizzy/ light-headed)**				

Severity grading for local and systemic AEs

GRADE 0	None
GRADE 1	Mild: Transient or mild discomfort (< 48 hours); No interference with activity; No medical intervention/therapy required
GRADE 2	Moderate: Mild to moderate limitation in activity – some assistance may be needed; no or minimal medical intervention/therapy required
GRADE 3	Severe: Marked limitation in activity, some assistance usually required; medical intervention/therapy required.
GRADE 4	Potentially Life-threatening: Requires assessment in A&E or hospitalization

Adverse Events of Special Interest

**Immunogenic**	Anaphylaxis
**Neurological**	Isolated anosmia/ageusia*<br>Guillain-Barre Syndrome<br>Acute disseminated encephalomyelitis (ADEM)<br>Aseptic meningitis<br>Meningoencephalitis<br>Peripheral facial nerve palsy<br>Generalised convulsion<br>Myelitis
**Hematological**	Thrombosis**<br>Stroke<br>Thrombocytopaenia***<br>Eosinophilia****<br>Coagulation disorder (includes coagulopathy, thrombosis, thromboembolism, internal/external bleed and stroke)
**Cardiac**	Acute cardiovascular injury (includes myocarditis, pericarditis, arrhythmias, heart failure, infarction)
**Dermatological**	Chilblain-like lesions<br>Erythema multiforme<br>Single organ cutaneous vasculitis<br>Alopecia
**Gastrointestinal**	Acute liver injury###<br>Appendicitis
**Respiratory**	ARDS##
**Renal**	Acute kidney injury
**Other**	COVID-19 disease#<br>SARS-COV-2 positivity on a validated test

*In the absence of COVID-19
** Excluding superficial thrombophlebitis (including line-associated)
*** G3 or above
**** This will be used as a marker of skewed Th2 responses and will be monitored in participants attending follow-up visits. Only G2 and above.
# In particular, any occurrence of suspected vaccine associated enhanced disease (VAED) as

CONSORT 2010 checklist of information to include when reporting a randomised trial*

Section/Topic	Item No	Checklist item	Reported on page No
**Title and abstract**			
	1a	Identification as a randomised trial in the title	1
	1b	Structured summary of trial design, methods, results, and conclusions (for specific guidance see CONSORT for abstracts)	3
**Introduction**			
Background and objectives	2a	Scientific background and explanation of rationale	4,5
	2b	Specific objectives or hypotheses	4,5
**Methods**			
Trial design	3a	Description of trial design (such as parallel, factorial) including allocation ratio	15,16
	3b	Important changes to methods after trial commencement (such as eligibility criteria), with reasons	NA
Participants	4a	Eligibility criteria for participants	15,16
	4b	Settings and locations where the data were collected	15,16
Interventions	5	The interventions for each group with sufficient details to allow replication, including how and when they were actually administered	15
Outcomes	6a	Completely defined pre-specified primary and secondary outcome measures, including how and when they were assessed	15,16
	6b	Any changes to trial outcomes after the trial commenced, with reasons	NA
Sample size	7a	How sample size was determined	19,20
	7b	When applicable, explanation of any interim analyses and stopping guidelines	21
Randomisation:			
Sequence generation	8a	Method used to generate the random allocation sequence	16,17
	8b	Type of randomisation; details of any restriction (such as blocking and block size)	16,17
Allocation concealment mechanism	9	Mechanism used to implement the random allocation sequence (such as sequentially numbered containers), describing any steps taken to conceal the sequence until interventions were assigned	16,17
Implementation	10	Who generated the random allocation sequence, who enrolled participants, and who assigned participants to interventions	16,17
Blinding	11a	If done, who was blinded after assignment to interventions (for example, participants, care providers, those	16,17

		assessing outcomes) and how	
	11b	If relevant, description of the similarity of interventions	16,17
Statistical methods	12a	Statistical methods used to compare groups for primary and secondary outcomes	20
	12b	Methods for additional analyses, such as subgroup analyses and adjusted analyses	20
**Results**			
Participant flow (a diagram is strongly recommended)	13a	For each group, the numbers of participants who were randomly assigned, received intended treatment, and were analysed for the primary outcome	5,6,Figure 1 (diagram)
	13b	For each group, losses and exclusions after randomisation, together with reasons	NA
Recruitment	14a	Dates defining the periods of recruitment and follow-up	20,21
	14b	Why the trial ended or was stopped	NA
Baseline data	15	A table showing baseline demographic and clinical characteristics for each group	Supplementary Table 1
Numbers analysed	16	For each group, number of participants (denominator) included in each analysis and whether the analysis was by original assigned groups	5,6
Outcomes and estimation	17a	For each primary and secondary outcome, results for each group, and the estimated effect size and its precision (such as 95% confidence interval)	6,7,8,9,Figures 2,3,5,6
	17b	For binary outcomes, presentation of both absolute and relative effect sizes is recommended	6,Figure 2
Ancillary analyses	18	Results of any other analyses performed, including subgroup analyses and adjusted analyses, distinguishing pre-specified from exploratory	7,8,Figure 4
Harms	19	All important harms or unintended effects in each group (for specific guidance see CONSORT for harms)	NA
**Discussion**			
Limitations	20	Trial limitations, addressing sources of potential bias, imprecision, and, if relevant, multiplicity of analyses	14
Generalisability	21	Generalisability (external validity, applicability) of the trial findings	14
Interpretation	22	Interpretation consistent with results, balancing benefits and harms, and considering other relevant evidence	11,12,14
**Other information**			
Registration	23	Registration number and name of trial registry	15,16
Protocol	24	Where the full trial protocol can be accessed, if available	Protocol provided in the Supplementary information
Funding	25	Sources of funding and other support (such as supply of drugs), role of funders	25

*We strongly recommend reading this statement in conjunction with the CONSORT 2010 Explanation and Elaboration for important clarifications on all the items. If relevant, we also recommend reading CONSORT extensions for cluster randomised trials, non-inferiority and equivalence trials, non-pharmacological treatments, herbal interventions, and pragmatic trials. Additional extensions are forthcoming: for those and for up to date references relevant to this checklist, see www.consort-statement.org.
