# Supplementary material for: A randomized controlled trial of heterologous ChAdOx1 nCoV-19 and recombinant subunit vaccine MVC-COV1901 against COVID-19
Source: Nat Commun. 2022 Sep 17;13:5466. doi: 10.1038/s41467-022-33146-7 (PMC9482645; doi:10.1038/s41467-022-33146-7)
Supplement: Supplementary file 3 — Reporting Summary [file 41467_2022_33146_MOESM3_ESM.pdf]

nature portfolio

Corresponding author(s): Kuan-Ying A. Huang

Last updated by author(s): Aug 31, 2022

Reporting Summary

Nature Portfolio wishes to improve the reproducibility of the work that we publish. This form provides structure for consistency and transparency in reporting. For further information on Nature Portfolio policies, see our Editorial Policies and the Editorial Policy Checklist.

Statistics

For all statistical analyses, confirm that the following items are present in the figure legend, table legend, main text, or Methods section.

n/a	Confirmed	
☐	☒	The exact sample size (*n*) for each experimental group/condition, given as a discrete number and unit of measurement
☐	☒	A statement on whether measurements were taken from distinct samples or whether the same sample was measured repeatedly
☐	☒	The statistical test(s) used AND whether they are one- or two-sided<br>*Only common tests should be described solely by name; describe more complex techniques in the Methods section.*
☒	☐	A description of all covariates tested
☒	☐	A description of any assumptions or corrections, such as tests of normality and adjustment for multiple comparisons
☐	☒	A full description of the statistical parameters including central tendency (e.g. means) or other basic estimates (e.g. regression coefficient) AND variation (e.g. standard deviation) or associated estimates of uncertainty (e.g. confidence intervals)
☐	☒	For null hypothesis testing, the test statistic (e.g. *F*, *t*, *r*) with confidence intervals, effect sizes, degrees of freedom and *P* value noted<br>*Give P values as exact values whenever suitable.*
☒	☐	For Bayesian analysis, information on the choice of priors and Markov chain Monte Carlo settings
☒	☐	For hierarchical and complex designs, identification of the appropriate level for tests and full reporting of outcomes
☐	☒	Estimates of effect sizes (e.g. Cohen's *d*, Pearson's *r*), indicating how they were calculated

Our web collection on statistics for biologists contains articles on many of the points above.

Software and code

Policy information about availability of computer code

Data collection	Trial Manager v 6
Data analysis	Prism v 9.1.1 (GraphPad), SAS v 9.4

For manuscripts utilizing custom algorithms or software that are central to the research but not yet described in published literature, software must be made available to editors and reviewers. We strongly encourage code deposition in a community repository (e.g. GitHub). See the Nature Portfolio guidelines for submitting code & software for further information.

Data

Policy information about availability of data

All manuscripts must include a data availability statement. This statement should provide the following information, where applicable:

- Accession codes, unique identifiers, or web links for publicly available datasets
- A description of any restrictions on data availability
- For clinical datasets or third party data, please ensure that the statement adheres to our policy

The data associated with this study are available within the article, its supplementary information and Source Data file. This trial is registered on ClinicalTrials.gov under the identifier NCT05054621. Source data are provided with this paper.

Field-specific reporting

Please select the one below that is the best fit for your research. If you are not sure, read the appropriate sections before making your selection.

☒ Life sciences ☐ Behavioural & social sciences ☐ Ecological, evolutionary & environmental sciences

For a reference copy of the document with all sections, see nature.com/documents/nr-reporting-summary-flat.pdf

Life sciences study design

All studies must disclose on these points even when the disclosure is negative.

Sample size	The primary objective of this trial was to determine if the immune response of heterologous group was non-inferior to that observed in homologous group, and the primary endpoints was neutralizing antibody titer at day 28 after booster vaccination. By assuming the non-inferiority margin was 0.67-fold-difference or -0.401 absolute difference of log geometric mean titers (GMT) between heterologous group and homologous group with the standard deviation 0.66, and the true difference of log GMT was 0, the study needed to recruit 44 evaluable participants per group (total 88 participants) to achieve 80% of power at one-sided 2.5% significance level. According to the missing rate 10% and the stratification in 1:1 for prime-boost 4-6 and 8-10 weeks, 50 participants for each stratum and equally random assigned to each group (25 for heterologous and 25 for homologous group) within strata (total 100 participants) is needed. The adjusted mean difference of log GMT was presented with the two-sided 95% confidence interval.
Data exclusions	All available safety and immunogenicity data at the time of the interim analysis time point were included.
Replication	No measurements were replicated due to invasiveness of the study.
Randomization	All eligible participants were 1:1 randomly assigned to receive a single dose of either the same vaccine as their prime dose ChAdOx1 (homologous group) or the Medigen COVID-19 vaccine MVC-COV1901 (heterologous group). Randomization was stratified by the intervals between prime and boost vaccination. Participants in each study arm were equally divided into two subgroups according to the intervals of 4-6 weeks and 8-10 weeks, respectively, between the prime and boost doses. The random list was generated by an independent study statistician using SAS software.
Blinding	The study was conducted in a single-blind fashion. Staffs involved in study delivery will be aware of what vaccine schedule the participant is receiving, the participant themselves will remain blinded to their booster vaccine. This blind will be maintained by applying a masking tape over the vaccine syringe. Laboratory staff will also be blinded to the vaccine schedule received.

Reporting for specific materials, systems and methods

We require information from authors about some types of materials, experimental systems and methods used in many studies. Here, indicate whether each material, system or method listed is relevant to your study. If you are not sure if a list item applies to your research, read the appropriate section before selecting a response.

Materials & experimental systems

n/a	Involved in the study	
☐	☒	Antibodies
☐	☒	Eukaryotic cell lines
☒	☐	Palaeontology and archaeology
☒	☐	Animals and other organisms
☐	☒	Human research participants
☐	☒	Clinical data
☒	☐	Dual use research of concern

Methods

n/a	Involved in the study	
☒	☐	ChIP-seq
☒	☐	Flow cytometry
☒	☐	MRI-based neuroimaging

Antibodies

Antibodies used	Secondary antibody used in the enzyme-linked immunospot assay:<br>1. alkaline phosphatase conjugated anti-human IgG antibody (polyclonal, Goat IgG)(Cat. No. 401442, Calbiochem, United States), 1:5000 dilution in sterile PBS<br>2. alkaline phosphatase conjugated anti-human IgM antibody (polyclonal, Goat IgG)(Cat. No. 401902, Calbiochem, United States), 1:5000 dilution in sterile PBS<br>3. alkaline phosphatase conjugated anti-human IgA antibody (polyclonal, Goat IgG)(Cat. No. 401132, Calbiochem, United States), 1:5000 dilution in sterile PBS<br>4. biotinylated anti-human IFN-γ antibody (7-B6-1 clone, Mouse IgG1)(Mabtech, United States), 1 μg/mL in sterile PBS with 5% BSA
Validation	All antibodies used were tested with appropriate negative and positive control samples. The information of all antibodies has been provided above and in the manuscript.<br>1. alkaline phosphatase conjugated anti-human IgG antibody (polyclonal, Goat IgG)(Cat. No. 401442, Calbiochem, United States):

https://www.sigmaaldrich.com/TW/en/product/mm/401442
2. alkaline phosphatase conjugated anti-human IgM antibody (polyclonal, Goat IgG)(Cat. No. 401902, Calbiochem, United States): https://www.sigmaaldrich.com/TW/en/product/mm/401902
3. alkaline phosphatase conjugated anti-human IgA antibody (polyclonal, Goat IgG)(Cat. No. 401132, Calbiochem, United States): https://www.sigmaaldrich.com/TW/en/product/mm/401132
4. biotinylated anti-human IFN-γ antibody (7-B6-1 clone, Mouse IgG1)(Mabtech, United States):
https://www.mabtech.com/products/anti-human-ifn-gamma-antibody-7-b6-1-biotin-3420-6
https://www.mabtech.com/products/elispot-flex-human-ifn-gamma-alp-3420-2a-0

Eukaryotic cell lines

Policy information about cell lines

Cell line source(s)	Vero cells (ATCC)
Authentication	Cell lines were frequently checked for cellular morphologies, growth rates and functions, but none of cell lines were authenticated.
Mycoplasma contamination	Cell lines were tested for mycoplasma and found to be mycoplasma-negative (MycoAlert Assay, Lonza and A2H 85011441, Sigma-Aldrich).
Commonly misidentified lines (See ICLAC register)	No commonly misidentified cell lines were used

Human research participants

Policy information about studies involving human research participants

Population characteristics	100 individuals were randomized assigned to receive ChAdOx1 (N = 50) or MVC-COV1901 (N = 50) as the booster dose at Chang Gung Memorial Hospital in Taiwan. All participants were taiwanese, 50% were male, and the mean age was 40.9 ± 8.8 years. Most of the participants were healthy adults without major systemic disorders. Type 2 diabetes and thyroid function disorders under medical control were respectively reported by three participants.
Recruitment	Participants were recruited by use of advertisement. Healthy adults aged 20–70 years who have no or well controlled comorbidities and their first dose of ChAdOx1 vaccine were eligible and recruited. For female participants, they must be either of non-childbearing potential (i.e., surgically sterilized or one year post-menopausal) or, if of childbearing potential, be abstinent or agree to use medically effective contraception on enrollment continuously until 90 days after boost immunization of study intervention. A negative pregnancy test was required before enrollment.<br>No potential biases were identified.
Ethics oversight	This study was approved by the Taiwan Food and Drug Administration and the ethics committee at Chang Gung Medical Foundation (Taiwan). The study was registered in ClinicalTrials.gov with ID NCT05054621 and the protocol in detail is available in Supplementary information.

Note that full information on the approval of the study protocol must also be provided in the manuscript.

Clinical data

Policy information about clinical studies
All manuscripts should comply with the ICMJE guidelines for publication of clinical research and a completed CONSORT checklist must be included with all submissions.

Clinical trial registration	NCT05054621
Study protocol	The protocol in detail is available with this publication as part of the supplementary file.
Data collection	The study took place at Chang Gung Memorial Hospital in Taiwan. Participant's screenings and recruitment began in August 2021. The earliest vaccination campaign began in September 2021.
Outcomes	Study objectives and outcomes are described within the study protocol. The primary objective of this trial was to determine if the immune response of heterologous group was non-inferior to that observed in homologous group, and the primary endpoints was neutralizing antibody titer at day 28 after booster vaccination.<br>The second endpoint was the safety of heterologous & homologous prime-boost immunization of vaccines. The solicited adverse events (AEs) occurring locally or systemically were assessed for 7 days following each vaccination from day 0 through day 7. Unsolicited AEs were recorded for 28 days after the boost dose. Serious AEs (SAEs) were recorded from signing of the informed consent form through day 168. Adverse events of special interest (AESIs) were recorded from the booster vaccination through day 168. For safety analysis, the number (%) of subjects with adverse events will be reported. Frequency counts and percentages will also be presented of subjects with serious adverse events, adverse events leading to withdrawal, adverse events by severity and adverse events by relationship to study treatment.
